# Temporal and regional trends of choking injuries in children in Italy, 2001–2013

**DOI:** 10.1186/s40621-018-0160-0

**Published:** 2018-08-01

**Authors:** Giulia Lorenzoni, Danila Azzolina, Nicola Soriani, Marco Galadini, Flavia Carle, Dario Gregori

**Affiliations:** 1 Unit of Biostatistics, Epidemiology and Public Health, Department of Cardiac, Thoracic and Vascular Sciences, University of Padova, Via Loredan, 18, 35121 Padova, Italy; 2 Directorate of Health Care Planning, Ministry of Health, Rome, Italy; 3 Centre of Epidemiology, Biostatistics and Information Technology, Università Politecnica delle Marche, Ancona, Italy

**Keywords:** Choking injuries, Children, Epidemiology, Italy, Hospitalizations

## Abstract

**Background:**

Choking injuries in children are a significant public health problem. The present study was aimed at examining the epidemiologic patterns of choking injuries in children using Italian official data from hospital discharge records.

**Methods:**

Hospital discharge records (from 2001 to 2013) reporting cases of choking injuries corresponding to the ICD-9 CM codes 933 (“Foreign body in pharynx and larynx”), 934 (“Foreign body in trachea, bronchus, and lung”), and fourth digit specifications (933.0 “pharynx”, 933.1 “larynx”, 934.0 “trachea”, 934.1 “main bronchus”, 934.8 “other specified parts”, 934.9 “respiratory tree, unspecified”) occurred in children aged 0–14 years were analyzed to assess the temporal and regional trends. Annual rates of hospitalizations due to choking injuries per 100,000 person-years were calculated and compared between boys and girls.

**Results:**

During the 13-year study period, there were a total of 7143 hospitalizations due to choking injuries in Italian children. The annual rates of hospitalizations due to choking injuries per 100,000 person-years decreased from 5.28 in 2001 to 3.46 in 2013 (*p* < 0.001). The reduction in choking injuries occurred across all the regions, particularly in Campania, Lombardia, Puglia, and Veneto.

**Conclusions:**

Hospitalizations for choking injuries in Italian children have decreased significantly in recent years. Choking injuries in children remain a cause of concern in some regions. Future research needs to elicit the causal factors underlying the downward trends and regional variations and develop targeted interventions to further reduce choking injuries in Italian children.

**Electronic supplementary material:**

The online version of this article (10.1186/s40621-018-0160-0) contains supplementary material, which is available to authorized users.

## Background

Among unintentional injuries, choking represents the leading cause of death in infants but remains relevant until the age of 14.

Old estimates suggest that 10,000 choking episodes occur every year in European Countries, of these 500 are fatal (RPA, 2003). However, it’s difficult to have precise and updated estimates of the overall burden of choking injuries, since the major source for such information, the European Injury Data Base (IDB), is far from being a complete source of information to estimate the epidemiology of this phenomenon, last but not least because only a few countries are represented (Keall et al., 2011). Other sources of information, like the Susy Safe database (Gregori, 2006), although wide in data collection, are not targeted to the estimation of incidence at EU or local level but in characterizing objects causing the injury and are therefore largely incomplete. Additionally, data on choking injuries generally come from hospital discharge records, not including self-resolved episodes and those referred to the Emergency Departments (without resulting in hospitalization). Using the network scale-up method (an estimator for hidden or difficult-to-reach populations (Snidero et al., 2004)), based on a CATI survey on 1081 Italian women, it has been found that only one out of eighty children reported to suffer from a choking injury were hospitalized (Snidero et al., 2012), thus demonstrating that the overall rate for choking injuries in pediatric ages is highly underestimated. This type of injuries is also related to relevant health care expenditure. A study evaluating the direct cost of Foreign Bodies (FB) injuries in children has shown that the higher costs are represented by choking injuries compared with FB in the digestive tract in the ear and in the nose. It has been estimated that the mean direct costs for managing FB in the pharynx and larynx is 800.74 €, for FB in trachea, bronchi and lungs is 1308 € (Gregori et al., 2007).

Taking into account the relevant public health burden associated with this phenomenon, it’s crucial to develop evidence-based policies to prevent choking episodes in children. Epidemiological surveillance is necessary to understand patterns of choking injuries in children, identifying items most frequently involved, most dangerous ones (e.g., marbles and coins are the most incident items, but magnets (Brown et al., 2014a) and batteries (Buttazzoni et al., 2015) cause the most severe complications), and predictors of choking episodes. However, despite the relevance of this phenomenon from the public health perspective, it is often neglected (Gregori et al., 2011; Passali et al., 2015). Generally, studies in this field are retrospective, single-center, case series (Brown et al., 2014b; Sahadan et al., 2011) or case studies (Chan et al., 2014; Nivatvongs et al., 2015), but a systematic collection of data on choking injuries is lacking. In this framework, one of the few examples of systematic data collection on FB injuries is represented by the Susy Safe registry (Gregori, 2006). It is one of the largest international databases recording cases of FB injuries (corresponding to the International Classification of Diseases, 9th revision, Clinical Modification, (ICD-9 CM) codes from 930 to 939) in children aged 0–14 years.

Data from Susy Safe registry interestingly showed that injury patterns are associated with the socio-cultural context in which the child lives, which inevitably influences families’ lifestyles. Furthermore, it is widely known that food represents the most important cause of choking in children (van As et al., 2012), independently from the socio-cultural environment. Such phenomenon depends on the psycho-physiological characteristics of kids that make them more prone than adults, to choke on specific food items sharing particular characteristics of shape, size, and texture. The most dangerous foods are internationally recognized to be those small and hard, such as nuts and seeds (Sih et al., 2012). However, the type of seeds on which children choke differs according to the cultural environment, which influences eating habits. A study has shown that U.S. children choke most often on sunflower seeds, while in other countries on watermelon ones (Kaushal et al., 2011). It has been demonstrated that in Finland, where fish consumption is widespread, most of FB episodes are due to fish bones (Gregori et al., 2008). Considering the variability of choking injuries’ characteristics related to the socio-cultural environment in which the child lives, it’s important to study patterns of choking injuries in specific countries to develop ad hoc prevention policies.

To our knowledge, no studies have systematically recorded and analyzed data on choking in Italian children with complete national coverage.

The study aimed at analyzing the epidemiology of choking injuries in children using Italian official data from hospital discharge records.

## Methods

Hospital discharge records (from 2001 to 2013) reporting cases of choking injuries corresponding to the ICD-9 CM codes 933 (“Foreign body in pharynx and larynx”), 934 (“Foreign body in trachea, bronchus, and lung”), and fourth digit specifications (933.0 “pharynx”, 933.1 “larynx”, 934.0 “trachea”, 934.1 “main bronchus”, 934.8 “other specified parts”, 934.9 “respiratory tree, unspecified”) occurred in children aged 0–14 years were analyzed.

Hospital discharge records represent a mandatory systematic data collection of hospitalizations that must be performed from all Italian hospitals facilities (with both public and private bodies). Records report both administrative and clinical information, representing a unique source of data to monitor the quality of care, and perform epidemiological research with the purpose of health planning. Selected data (to protect anonymity) reported in the records could be requested from Italian Ministry of Health, Directorate of Health Care Planning, in the context of a research project.

The information on hospitalizations regarded patients’ demographic characteristics (age, sex, and residence), length of stay in the hospital, main and secondary diagnosis at the discharge, procedures performed, Diagnosis Related Group (DRG), type of discharge (e.g., to home, to nursing home, discharge against medical advice).

The analysis of the epidemiology of this phenomenon in Italian children was done by estimating the rates of hospitalizations due to choking for each Region from 2001 to 2013, and an overview of the characteristics of children hospitalized, and of the hospitalizations has been provided.

### Statistical analysis

Descriptive statistics of data according to children age groups was performed. Data were reported as median (I and III quartiles) for continuous variables, and percentages (absolute numbers), for qualitative variables. Wilcoxon-Kruskal-Wallis test was performed for continuous variables and Pearson chi square test for categorical ones.

Yearly rates (95% Confidence Interval) of choking injuries were computed using data on resident population in each Italian’s region from 2001 to 2013 (demo ISTAT G, 2015). The estimates were obtained using a normal approximation confidence interval for absolute rates. Confidence intervals for rate ratios were obtained using a Negative Binomial Regression model for hospitalization event (Hilbe, 2011).

A Mann-Kendall Trend Test was performed to test if a monotonic trend existed in hospitalization time series for male, female, and overall hospitalizations.

Statistical analyses were performed using R system (Team R. R Development Core Team, 2013), *rms* package (Harrell Jr, 2013) and *lsmeans* library (Harrell Jr, 2013).

## Results

Hospitalizations due to choking injuries between 2001 and 2013 were 7143, corresponding to an overall hospitalization rate of 6.87 per 100,000 person-years. The number of hospitalizations was higher in infants aged 0–12 months (3744) and in toddlers aged 1–3 years (2380) compared with children and adolescents aged 4–14 (1019). Specifically, the hospitalization rate ratio for infants aged 0–12 compared to 4–14 was 36.26 (28.36; 46.38 95% C.I.), while for toddlers 1–3 years was 8.63 (6.74; 11.04 95% C.I.). No hospitalizations due to choking were reported from a few regions in some years, particularly from Molise and Valle d’Aosta.

Yearly national hospitalization rates ranged from 10.42 per 100,000 person-years (9.73; 11.5 95% C.I.) in 2002 to 3.46 per 100,000 person-years (3.08; 3.89, 95% C.I.) in 2013. They were slightly higher in male compared to female children (Table 1). Specifically, considering the overall period under consideration, the hospitalization rate ratio for male compared to female was 1.31 (1.08; 1.60, 95% C.I.).

**Table 1:** National hospitalization (95% Confidence Interval) rates in children (over 100,000 person-years)

	Male children	Female children	Overall
2001	7.5 (94); [6.09,9.22]	6.33 (75); [5.01,7.98]	5.28 [4.8;5.81]
2002	5.09 (31); [3.52,7.32]	3.17 (18); [1.93,5.11]	10.42 [9.73;11.15]
2003	7.1 (154); [6.04,8.34]	4.85 (100); [3.97,5.93]	9.05 [8.41;9.73]
2004	4.9 (349); [4.41,5.45]	4.26 (289); [3.79,4.79]	8.89 [8.26;9.56]
2005	5.66 (219); [4.94,6.47]	4.5 (164); [3.85,5.26]	7.93 [7.34;8.57]
2006	5.57 (59); [4.28,7.24]	3.8 (38); [2.72,5.27]	7.27 [6.7;7.88]
2007	5.7 (310); [5.09,6.38]	5.29 (272); [4.68,5.96]	7.73 [7.15;8.35]
2008	5.24 (67); [4.09,6.7]	3.89 (47); [2.89,5.21]	5.86 [5.35;6.4]
2009	4.27 (405); [3.87,4.72]	3.74 (334); [3.35,4.16]	5.48 [5;6.01]
2010	6.34 (92); [5.14,7.82]	6.68 (91); [5.41,8.24]	5.57 [5.08;6.1]
2011	4.63 (14); [2.63,7.98]	2.8 (8); [1.3,5.76]	4.89 [4.43;5.39]
2012	3.26 (128); [2.73,3.89]	2.56 (95); [2.08,3.14]	4.5 [4.07;4.99]
2013	11.3 (511); [10.35,12.34]	11.22 (480); [10.25,12.28]	3.46 [3.08;3.89]

**Fig. 1:**
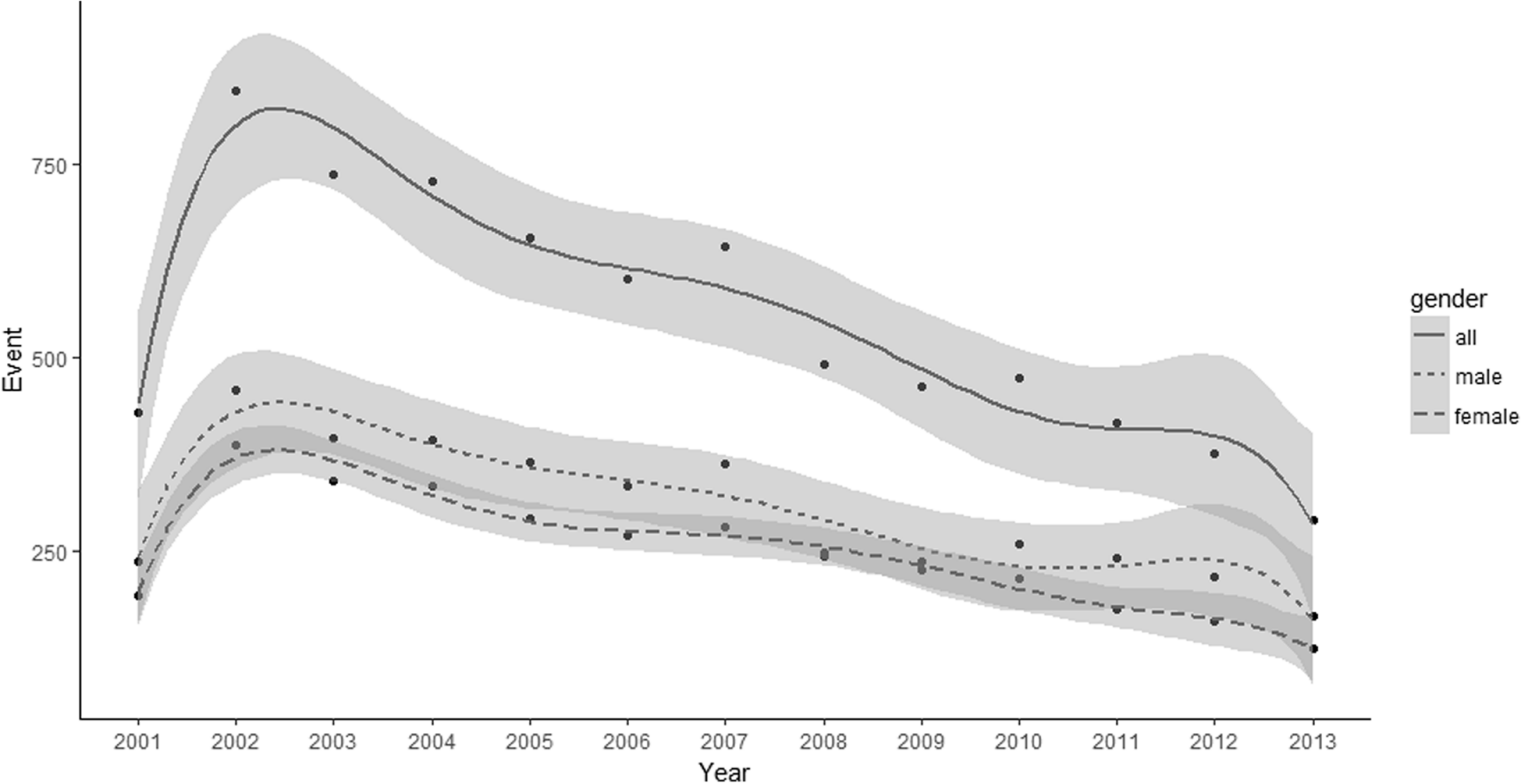
National hospitalizations time trends. Data were interpolated considering a linear model smoothing procedure with a polynomial approximation consisting in 6 degrees. The y-axis reports the hospitalizations count

**Fig. 2:**
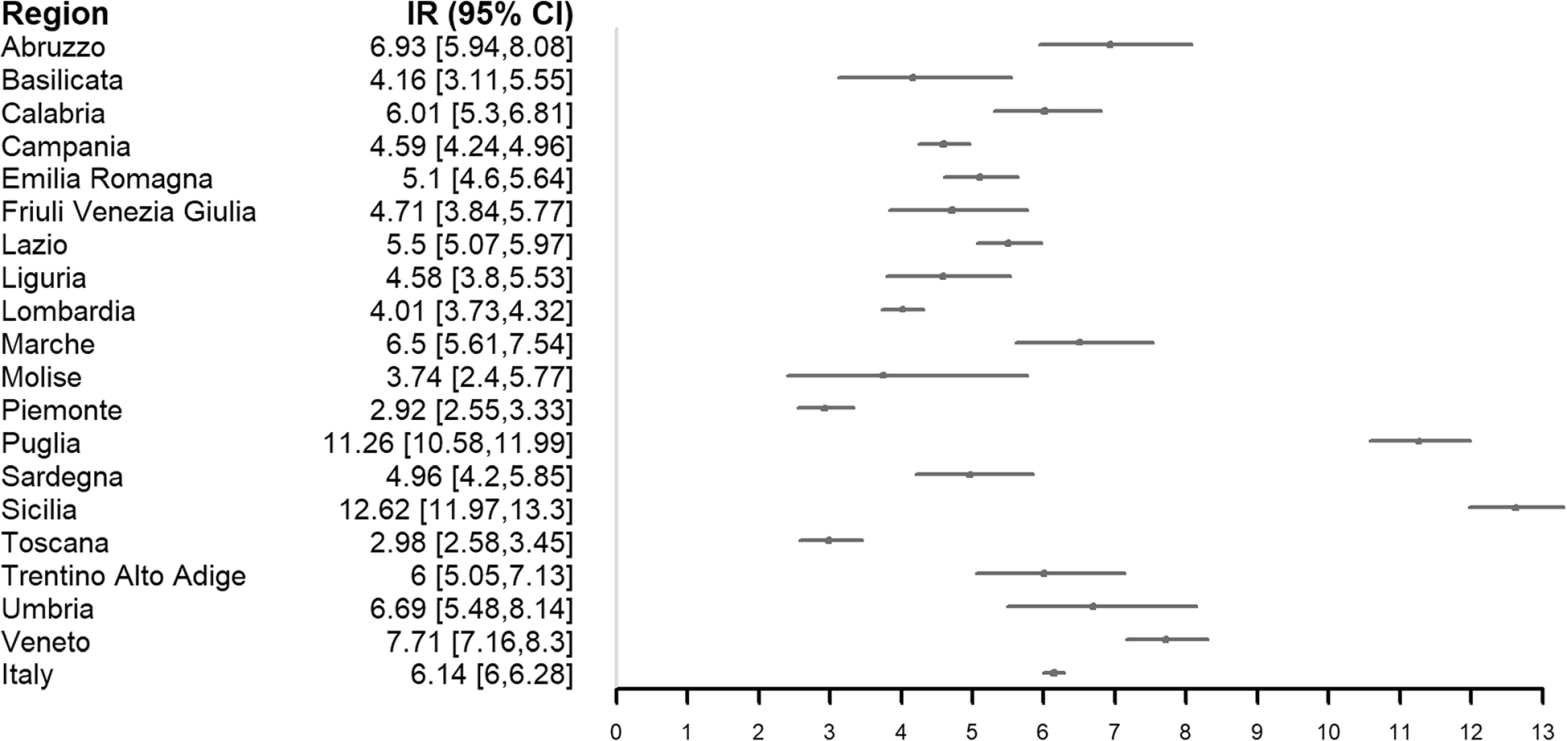
Confidence Interval (95%) plot of Hospitalization incidence rate by region

Considering the national hospitalizations (overall and according to children’s gender), the analysis of time trends showed a significant decrease of hospitalization overall (*p*-value < 0.001), in male children (*p*-value 0.002) and female ones (*p*-value < 0.001) (Fig. 1). The significant decreasing pattern in the hospitalization trend was also confirmed for many Italian regions as shown in Table 2. Looking specifically at the hospitalization rates, some regions (particularly Sicilia and Puglia) showed significantly higher rates of hospitalization compared with the other ones, both overall (Fig. 2) and stratified by year (Table 3). Looking at the gender, no significant differences were found in hospitalizations rate ratios stratified by region (Additional file 1: Table S1), even if regional rates of hospitalization seemed to be slightly higher in boys than in girls (Additional file 1: Tables S2 and S3).

**Table 2:** Kendall-Tau hospitalization trend test statistics by Region

Region	Kendall-Tau	se (Tau)	*P*-Value
Abruzzo	− 0.55	16.73	0.01
Basilicata	−0.34	14.96	0.16
Calabria	−0.52	16.64	0.02
Campania	−0.85	16.64	< 0.01
Emilia Romagna	−0.52	14.84	0.03
Friuli Venezia Giulia	−0.08	17.98	0.78
Lazio	−0.47	16.8	0.03
Liguria	−0.27	15.29	0.27
Lombardia	−0.79	14.87	< 0.01
Marche	−0.59	16.67	0.01
Molise	−0.65	11.1	0.02
Piemonte	−0.3	15.19	0.21
Puglia	−0.79	16.63	< 0.01
Sardegna	−0.4	16.72	0.07
Sicilia	−0.56	16.77	0.01
Toscana	−0.36	15.22	0.11
Trentino Alto Adige	−0.45	14.74	0.06
Umbria	−0.4	16.74	0.07
Veneto	−0.74	14.82	< 0.01

**Table 3:** Regional hospitalization (95% Confidence Interval) rates in children (over 100,000 person-years). Aggregated data have been provided for reasons of data disclosure for some regions (Calabria-Basilicata; Molise-Marche-Umbria; Piemonte-Valle d’Aosta)

	2001	2002	2003	2004	2005	2006	2007	2008	2009	2010	2011	2012	2013
Abruzzo	10.14 (18) [6.2,16.38]	10.8 (19) [6.69,17.22]	12.52 (22) [8.04,19.3]	8.55 (15) [4.97,14.46]	7.4 (13) [4.12,13.03]	3.43 (6) [1.39,7.88]	10.95 (19) [6.78,17.45]	9.76 (17) [5.87,15.99]	5.73 (10) [2.91,10.92]	4.01 (7) [1.76,8.67]	4.58 (8) [2.13,9.42]	5.3 (9) [2.59,10.46]	3.52 (6) [1.43,8.09]
Basilicata Calabria	8.68 (38) [6.23,12.05]	6.07 (26) [4.05,9.04]	6.46 (27) [4.34,9.53]	6.85 (28) [4.64,10.04]	6.49 (26) [4.33,9.66]	7.14 (28) [4.84,10.47]	5.99 (23) [3.89,9.15]	5.3 (20) [3.33,8.34]	4.03 (15) [2.34,6.82]	6.52 (24) [4.27,9.86]	7.68 (28) [5.2,11.26]	4.24 (15) [2.46,7.17]	1.43 (5) [0.53,3.54]
Campania	8.5 (91) [6.88,10.49]	6.82 (72) [5.38,8.65]	5.83 (61) [4.49,7.54]	6.65 (69) [5.21,8.47]	6.33 (65) [4.92,8.12]	4.63 (47) [3.44,6.21]	5.4 (54) [4.1,7.11]	4.46 (44) [3.28,6.05]	2.47 (24) [1.62,3.73]	3.84 (37) [2.74,5.36]	4.3 (41) [3.12,5.89]	2.04 (19) [1.27,3.26]	1.52 (14) [0.87,2.62]
Emilia Romagna	–	10.11 (47) [7.51,13.56]	7.51 (36) [5.33,10.51]	7.3 (36) [5.19,10.23]	4.71 (24) [3.09,7.13]	5.17 (27) [3.48,7.64]	7.3 (39) [5.26,10.09]	6.39 (35) [4.52,8.99]	5.85 (33) [4.09,8.32]	4.66 (27) [3.13,6.88]	4.41 (26) [2.94,6.56]	4.62 (27) [3.11,6.83]	4.39 (26) [2.93,6.53]
Friuli Venezia Giulia	–	3.67 (5) [1.35,9.11]	10.08 (14) [5.74,17.37]	3.55 (5) [1.31,8.8]	6.29 (9) [3.07,12.41]	3.44 (5) [1.27,8.54]	11.55 (17) [6.95,18.92]	6.02 (9) [2.94,11.87]	5.25 (8) [2.44,10.8]	3.89 (6) [1.58,8.94]	2.57 (4) [0.82,7.08]	4.55 (7) [1.99,9.83]	5.16 (8) [2.4,10.61]
Lazio	9.29 (66) [7.24,11.9]	7.48 (53) [5.66,9.86]	5.6 (40) [4.05,7.71]	7.75 (56) [5.91,10.15]	7.64 (56) [5.83,10]	6.11 (45) [4.51,8.25]	7.21 (55) [5.48,9.46]	4.53 (35) [3.2,6.37]	3.69 (29) [2.52,5.38]	3.78 (30) [2.6,5.48]	4.75 (38) [3.41,6.59]	5.26 (40) [3.81,7.23]	5.06 (39) [3.65,6.99]
Liguria	–	5.97 (10) [3.04,11.38]	9.44 (16) [5.59,15.71]	7.01 (12) [3.8,12.62]	4.59 (8) [2.14,9.44]	5.62 (10) [2.85,10.7]	3.9 (7) [1.71,8.42]	3.32 (6) [1.35,7.63]	3.82 (7) [1.67,8.25]	3.25 (6) [1.32,7.46]	5.91 (11) [3.11,10.93]	7.72 (14) [4.4,13.31]	3.85 (7) [1.69,8.31]
Lombardia	–	7.63 (91) [6.18,9.41]	7.16 (87) [5.77,8.88]	5.73 (71) [4.51,7.27]	5.04 (64) [3.92,6.48]	5.11 (66) [3.98,6.54]	5.17 (68) [4.05,6.6]	3.96 (53) [3,5.22]	3.96 (54) [3,5.21]	4.25 (59) [3.26,5.52]	3.26 (46) [2.41,4.39]	3.46 (48) [2.58,4.63]	2.28 (32) [1.59,3.26]
Marche Molise Umbria	10.4 (35) [7.36,14.64]	12.15 (41) [8.83,16.65]	6.75 (23) [4.38,10.3]	8.43 (29) [5.75,12.28]	8.62 (30) [5.92,12.47]	8.55 (30) [5.87,12.37]	5.09 (18) [3.11,8.23]	6.16 (22) [3.96,9.49]	4.14 (15) [2.41,7.01]	6.88 (25) [4.55,10.33]	3.56 (13) [1.98,6.26]	3.61 (13) [2.01,6.34]	3.88 (14) [2.21,6.68]
Piemonte V.d’Aosta	–	2.86 (15) [1.66,4.83]	5.07 (27) [3.41,7.49]	3.52 (19) [2.18,5.61]	5.08 (28) [3.44,7.45]	2.88 (16) [1.7,4.79]	4.99 (28) [3.38,7.32]	2.63 (15) [1.53,4.44]	3.27 (19) [2.03,5.22]	3.41 (20) [2.14,5.37]	3.21 (19) [1.99,5.12]	1.89 (11) [0.99,3.49]	1.7 (10) [0.87,3.24]
Puglia	16.47 (112) [13.62,19.9]	19.4 (130) [16.27,23.11]	13.91 (92) [11.27,17.14]	13.32 (87) [10.73,16.51]	12.78 (83) [10.24,15.93]	17.32 (111) [14.31,20.94]	13.8 (87) [11.12,17.11]	9.63 (60) [7.41,12.48]	8.61 (53) [6.52,11.36]	10.32 (63) [8,13.3]	7.77 (47) [5.77,10.43]	5.41 (32) [3.76,7.73]	5.81 (34) [4.09,8.22]
Sardegna	5.65 (13) [3.14,9.94]	6.2 (14) [3.53,10.68]	4.49 (10) [2.28,8.56]	5.48 (12) [2.97,9.86]	7.87 (17) [4.74,12.89]	10.8 (23) [7.01,16.49]	7.14 (15) [4.15,12.07]	5.76 (12) [3.12,10.38]	1.93 (4) [0.62,5.31]	1.94 (4) [0.62,5.34]	5.84 (12) [3.16,10.51]	3.49 (7) [1.53,7.54]	2 (4) [0.64,5.5]
Sicilia	6.47 (56) [4.93,8.46]	21.32 (181) [18.38,24.72]	17.5 (147) [14.84,20.63]	18.86 (157) [16.07,22.11]	16.54 (136) [13.93,19.63]	12.69 (103) [10.41,15.46]	16.27 (130) [13.65,19.39]	13.98 (110) [11.54,16.92]	14.65 (114) [12.14,17.67]	11.53 (89) [9.31,14.25]	9.53 (73) [7.52,12.05]	9.94 (74) [7.86,12.55]	5.01 (37) [3.58,6.99]
Toscana	–	5.37 (22) [3.45,8.28]	6.49 (27) [4.36,9.59]	5.91 (25) [3.9,8.86]	3.7 (16) [2.19,6.16]	1.82 (8) [0.85,3.75]	5.38 (24) [3.53,8.15]	1.98 (9) [0.97,3.91]	2.16 (10) [1.1,4.11]	2.55 (12) [1.38,4.59]	1.26 (6) [0.51,2.89]	3.19 (15) [1.85,5.39]	2.73 (13) [1.52,4.8]
Trentino Alto Adige	–	13.87 (21) [8.81,21.61]	11.09 (17) [6.68,18.17]	5.81 (9) [2.83,11.46]	6.36 (10) [3.23,12.11]	11.94 (19) [7.4,19.04]	4.37 (7) [1.91,9.43]	5.57 (9) [2.72,10.99]	6.13 (10) [3.11,11.68]	6.1 (10) [3.1,11.62]	4.24 (7) [1.86,9.16]	6.11 (10) [3.11,11.65]	3.05 (5) [1.12,7.58]
Veneto	–	16.01 (98) [13.07,19.6]	14.58 (91) [11.81,17.99]	15.44 (98) [12.6,18.91]	10.81 (70) [8.49,13.75]	8.82 (58) [6.75,11.48]	7.79 (52) [5.88,10.3]	5.01 (34) [3.53,7.09]	8.26 (57) [6.31,10.78]	7.6 (53) [5.75,10.02]	5.27 (37) [3.76,7.34]	4.91 (34) [3.45,6.95]	5.04 (35) [3.56,7.09]

Table 4 shows the main characteristics of the hospitalizations stratified according to the age groups (infants, toddlers, and older children). The female gender was markedly less prevalent among infants compared to the other age groups (*p*-value < 0.001). Almost all of the children were Italian (93%), the other most represented nationalities (considering sample as a whole) were the Albanian (1.1%), the Romanian (1.2%), the Moroccan (1.4%), and the Tunisian (0.3%). The distribution of nationalities reflects the main migration flows in Italy.

**Table 4:** Characteristics of hospitalizations according to age groups (3744 hospitalizations in infants, 2380 hospitalizations in toddlers, 1019 hospitalizations in older children). Data are percentages (absolute numbers)

		Hospitalizations in infants	Hospitalizations in toddlers	Hospitalizations in older children	Hospitalizations overall	*P*-value
		(*N* = 3744)	(*N* = 2380)	(*N* = 1019)	(*N* = 7143)	
*Gender: Female*	7143	1% (1924)	39% (924)	38% (385)	45% (3233)	< 0.001
*Nationality: Other*	7137	2% (65)	4% (98)	4% (39)	3% (202)	< 0.001
*Italian*		5% (3564)	89% (2127)	93% (950)	93% (6641)	
*Albania*		1% (37)	1% (32)	1% (13)	1% (82)	
*Romania*		1% (22)	3% (61)	0% (5)	1% (88)	
*Marocco*		1% (40)	2% (52)	1% (7)	1% (99)	
*Tunisia*		0% (13)	0% (9)	0% (3)	0% (25)	
*Hospitalization type: newborn*	7003	0% (1)	0% (0)	0% (1)	0% (2)	0.017
*ordinary hospitalization*		8% (299)	9% (209)	11% (103)	9% (611)	
*urgent hospitalization*		92% (3391)	90% (2104)	89% (860)	91% (6355)	
*programmed with pre-hospitalization*		0% (5)	0% (0)	0% (1)	0% (6)	
*Other*		0% (10)	1% (16)	0% (3)	0% (29)	
*Day Hospital*	7143	1% (26)	2% (52)	6% (64)	2% (142)	< 0.001
*Hospitalization days*	7143	1/2/3	1/2/3	1/1/2	1/2/3	< 0.001
*Discharge*: *Death*	7143	0% (5)	1% (12)	0% (5)	0% (22)	< 0.001
*Ordinary discharge at home*		84% (3148)	81% (1935)	84% (857)	83% (5940)	
*Ordinary discharge in healthcare residence*		0% (0)	0% (4)	0% (1)	0% (5)	
*Home hospitalization*		1% (19)	1% (13)	0% (5)	1% (37)	
*Discharged against medical advice*		14% (516)	9% (226)	11% (107)	12% (849)	
*Transferred another hospital*		1% (54)	8% (183)	4% (42)	4% (279)	
*Transferred same hospital*		0% (1)	0% (7)	0% (1)	0% (9)	
*Rehabilitation*		0% (1)	0% (0)	0% (1)	0% (2)	

Regarding the characteristics of hospitalizations, in most cases, independently from age group, an inpatient urgent hospitalization was performed (day hospital services represented only 142 hospitalizations). The median length of stay (of regular inpatient hospitalizations) was slightly longer for infants and toddlers (2 days) compared to 1 day of older children (*p*-value < 0.001). Most children were discharged to home (83%), and only a few were discharged against medical advice (12%). Twenty-two children, 3.08‰, died (1.98‰; 4.74‰, 95% C.I.), most of them were toddlers (2.73‰; 9.06‰, 95% C.I.). Looking at the hospitalization characteristics among each age group and stratified according to gender (Additional file 1: Tables S4 to S9), generally girls were younger than boys. Among infants, the median age of baby girls was 38 days and that of baby boys was 44 days. Among toddlers, slightly most females were 1 year of age compared to males. The median age of older children was of 6 years in girls and of 7 years in boys (p-value < 0.001). The median length of stay was of 2 days for infants and toddlers of both gender, while it was lower (median of 1 day) in older children. The main procedures most frequently performed differed according to age group, especially in infants. Toddlers and older children underwent more often to fiber-optic bronchoscopy, removal of the intraluminal foreign body from pharynx, trachea, and bronchus without an incision. Conversely, the main procedures most frequently performed in infants were routine chest x-ray, electrocardiogram and microscopic examination of blood. Procedures for FB removal (such as bronchoscopy) were less frequent. This is probably related to the age of children (very young) and to the fact that the ICD codes searched include also choking on vomitus, mucus, and liquids.

## Discussion

The study aimed at analyzing the epidemiology of choking injuries in children, using Italian data from hospital discharge records. To our knowledge, this is one of the few studies that has analyzed the trend in hospitalizations for choking injuries in children with national coverage.

Consistently with literature, the hospitalizations were more prevalent in the youngest age group (Agran et al., 2003).

Interestingly, Italian rates of hospitalizations due to choking were found to decrease over the period considered. The improvement of the burden of hospitalizations for choking injuries in Italian children may be the result of coexisting factors. Such factors include the approval of the EU Toy Safety Directive 2009/48/EC (Snidero et al., 2007a) (that has established the safety criteria for toys marketed in EU) and the consecutively introduction of different versions of the ICD during the time period considered (ICD-9 CM 1997 Italian version from 2001 to 2005, ICD-9 CM 2002 Italian version from 2006 to 2008, and ICD-9 CM 2007 Italian version from 2009 and currently used) which may have led to changes in codification of diagnosis of interest (or of similar diagnosis). Additionally, it has been shown great variability in hospitalization rates among different regions which is in line with previous research in the field showing geographical heterogeneity in unintentional injuries’ incident rates in Italy (Snidero et al., 2007b). In some regions, a dramatically higher rate of hospitalizations due to choking injuries have been recorded compared to other geographical areas. Conversely, some regions (such as Molise and Valle d’Aosta) reported no hospitalizations in some years; thus requiring a more in-depth analysis of the phenomenon in these geographical areas, better understanding the reason why no hospitalizations have been reported. Taking into account the fact that Italian regions are in charge, at least partially, of health care planning and managing (Cicchetti & Gasbarrini, 2016), resulting in differences in regional health care organizations (Rosso et al., 2015), several explanations may be hypothesized to understand such cross regional variability in hospitalizations rate. It could be related to the fact that children who choke are more likely to be hospitalized in these geographical areas. Such heterogeneity may also result from the development of specific prevention programs in some regions, and the lack of such interventions in the other ones. Otherwise, it may be associated with an inappropriate coding of the diagnosis, leading to an overestimation of hospitalizations due to choking injuries in some areas or to an underestimation of this phenomenon in other regions (e.g., complications due to FB aspiration might be reported in the main diagnosis field if they represent the reason why the child has been hospitalized). However, it could indicate really that in some regions more children suffer from choking injuries. In this case, it’s crucial to deeply analyze this phenomenon to identify factors predicting choking injuries in these areas and developing ad hoc prevention policies.

Twenty-two deaths out of 7143 hospitalizations have been recorded and, in line with literature (Pearson & Stone, 2009), were more prevalent in children ages 0–3 years. Estimating mortality for choking is difficult due to problems with coding: generally choking is not reported as the main diagnosis when death occurred. Furthermore, considering only deaths resulting from hospitalizations (and not including those occurred in the Emergency Departments or outside the hospital) may lead to an underestimation of the phenomenon. It could be useful to study the mortality for choking injuries (and comparing the results with official data), using different research methods, such as the analysis of death cases due to choking reported from mass media (news reports particularly).

### Study limitations

Our study presents some limitations. First, injury rates are based exclusively on data about hospitalizations, not including cases referred to the Emergency Department without resulting in hospitalizations and of course, those self-resolved at home. A ten-year investigation on non-fatal food choking injuries in children aged 0–14 referred to Emergency Departments in the US showed that only 10% of injured children were hospitalized, suggesting that considering only hospitalizations for choking may lead to an underestimation of the phenomenon (Chapin et al., 2013). Unfortunately, a systematic uniform data collection of cases managed in the Emergency Departments did not exist in Italy until 2012 (Snidero et al., 2007a). Furthermore, the codes used for identifying choking in hospital discharge records (ICD 933 and 934) include also cases of aspiration of liquids, vomit, and mucus. These injuries are most frequently in the context of complicated clinical conditions (e.g., general anesthesia, trauma, severe disability), whose incidence is generally low. However, the type of procedures performed in infants may be susceptible of choking on vomitus or liquids, given the low frequency of procedures for FB removal (such as bronchoscopy). This issue may be overcome, at least partially, by analyzing the external cause codes, which have been recently added to the hospital discharge records, and by adopting the tenth version of the ICD (which is actually adopted in Italy only for the coding of the causes of death), which better specifies the diagnosis of choking injury, differentiating gastric contents and food from unspecified (or other) FB. Specific codes have been added, such as, “Gastric contents in pharynx” (T17.21), “Food in pharynx” (T17.22), “Gastric contents in larynx” (T17.31), “Food in larynx” (T17.32), the same for trachea, bronchi, and unspecified (or other) parts of the respiratory tract. More precise classification of choking injuries in hospital discharge records may improve epidemiological surveillance of this phenomenon, better characterizing choking episodes. Not least, since the present study was based on administrative data (i.e., hospital discharge records) information about FB characteristics were not available.

## Conclusions

National trends of hospitalizations for choking in children aged 0–14 years were found to decrease significantly over the period considered (2001–2013); however, relevant differences have been recorded among the hospitalization rates of Italian regions.

Present findings are relevant from the perspective of health planning. Further efforts should be put forward to understand the reasons of such heterogeneity in hospitalization rates, with the aim of planning and implementing ad hoc interventions acting upon factors on which such heterogeneity depends.

## Additional file

Additional file 1: **Table S1.** Hospitalization (95% Confidence Interval) Rate Ratio for male compared to female, estimated using a Negative Binomial. **Table S2.** Hospitalization (95% Confidence Interval) rate in male children (over 100,000 person-years). Aggregated data have been provided for reasons of data disclosure for some regions. **Table S3.** Hospitalization (95% Confidence Interval) rate in female children (over 100,000 person-years). Aggregated data have been provided for reasons of data disclosure for some regions. **Table S4.** Characteristics of hospitalization in male infants. **Table S5.** Characteristics of hospitalization in female infants. **Table S6.** Characteristics of hospitalization in male toddlers. **Table S7.** Characteristics of hospitalization in female toddlers. **Table S8.** Characteristics of hospitalization in male children (4–14 years). **Table S9.** Characteristics of hospitalization in female children (4–14 years)x`. (DOCX 73 kb)

## References

[CR1] Agran PF, Anderson C, Winn D, Trent R, Walton-Haynes L, Thayer S (2003). Rates of pediatric injuries by 3-month intervals for children 0 to 3 years of age. Pediatrics.

[CR2] Brown JC, Baik FM, Ou HC, Otjen JP, Parish HG, Chan DK (2014). Upper aerodigestive magnetic foreign bodies in children. Laryngoscope.

[CR3] Brown JC, Otjen JP, Drugas GT (2014). Pediatric magnet ingestions: the dark side of the force. Am J Surg.

[CR4] Buttazzoni E, Gregori D, Paoli B, Soriani N, Baldas S, Rodriguez H (2015). Symptoms associated with button batteries injuries in children: an epidemiological review. Int J Pediatr Otorhinolaryngol.

[CR5] Chan SS-C, Russell M, Ho-Fung VM (2014). Not all radiopaque foreign bodies shadow on ultrasound: unexpected sonographic appearance of a radiopaque magnet. Ultrasound quarterly.

[CR6] Chapin MM, Rochette LM, Annest JL, Haileyesus T, Conner KA, Smith GA (2013). Nonfatal choking on food among children 14 years or younger in the United States, 2001–2009. Pediatrics.

[CR7] Cicchetti A, Gasbarrini A (2016). The healthcare service in Italy: regional variability. Euro rev med pharmacological scie.

[CR8] demo ISTAT G. Demografia in cifre. Disponibile all’indirizzo http://demo.istat.it/index.html (ultimo accesso giugno 2015).

[CR9] Gregori D (2006). The Susy safe project. A web-based registry of foreign bodies injuries in children. Int J Pediatr Otorhinolaryngol.

[CR10] Gregori D, Foltran F, Passali D (2011). Foreign body injuries in children: need for a step forward against an old yet neglected epidemic. Paediatr Perinat Epidemiol.

[CR11] Gregori D, Salerni L, Scarinzi C, Morra B, Berchialla P, Snidero S (2008). Foreign bodies in the upper airways causing complications and requiring hospitalization in children aged 0–14 years: results from the ESFBI study. Eur Arch Otorhinolaryngol.

[CR12] Gregori D, Scarinzi C, Berchialla P, Snidero S, Rahim Y, Stancu A (2007). The cost of foreign body injuries in the upper aero-digestive tract: need for a change from a clinical to a public health perspective?. Int J Pediatr Otorhinolaryngol.

[CR13] Harrell FE (2013). rms: Regression Modeling Strategies. R package version 4.0–0. City.

[CR14] Hilbe JM (2011). Negative binomial regression.

[CR15] Kaushal P, Brown DJ, Lander L, Brietzke S, Shah RK (2011). Aspirated foreign bodies in pediatric patients, 1968–2010: a comparison between the United States and other countries. Int J Pediatr Otorhinolaryngol.

[CR16] Keall MD, Ormandy D, Baker MG (2011). Injuries associated with housing conditions in Europe: a burden of disease study based on 2004 injury data. Environ Health.

[CR17] Nivatvongs W, Ghabour M, Dhanasekar G (2015). Difficult button battery ear foreign body removal: the magnetic solution. J Laryngol Otolo.

[CR18] Passali D, Gregori D, Lorenzoni G, Cocca S, Loglisci M, Passali F (2015). Foreign body injuries in children: a review. Acta Otorhinolaryngol Ital.

[CR19] Pearson J, Stone DH (2009). Pattern of injury mortality by age-group in children aged 0–14 years in Scotland, 2002–2006, and its implications for prevention. BMC Pediatr.

[CR20] Rosso A, Marzuillo C, Massimi A, De Vito C, de Belvis AG, La Torre G (2015). Policy and planning of prevention in Italy: results from an appraisal of prevention plans developed by regions for the period 2010–2012. Health Policy.

[CR21] RPA (2003). Inedibles in food product packaging.

[CR22] Sahadan D, Zainudin N, Kassim A, Zahari Z, Mahadzir M, Daud C (2011). Case series of foreign body aspiration in Paediatric institute. Hosp Kuala Lumpur Med j Malaysia.

[CR23] Sih T, Bunnag C, Ballali S, Lauriello M, Bellussi L (2012). Nuts and seed: a natural yet dangerous foreign body. Int J Pediatr Otorhinolaryngol.

[CR24] Snidero S, Corradetti R, Gregori D (2004). The network scale-up method: a simulation study in case of overlapping sub-populations. Metodoloski zvezki.

[CR25] Snidero S, Morra B, Corradetti R, Gregori D (2007). Use of the scale-up methods in injury prevention research: an empirical assessment to the case of choking in children. Soc Networks.

[CR26] Snidero S, Rahim Y, Berchialla P, Gregori D (2007). Risk factors and geographical heterogeneity in unintentional home injuries incidence rate: new evidence based on Multiscopo survey in Italy. Int J Inj Control Saf Promot.

[CR27] Snidero S, Soriani N, Baldi I, Zobec F, Berchialla P, Gregori D (2012). Scale-up approach in CATI surveys for estimating the number of foreign body injuries in the aero-digestive tract in children. Int J Environ Res Public Health.

[CR28] Team R. R Development Core Team (2013). RA Lang Environ Stat Comput.

[CR29] van As ABS, Yusof AM, Millar AJ, Group SSW. Food foreign body injuries. Int J Pediatr Otorhinolaryngol. 2012;76:S20–S5. 10.1016/j.ijporl.2012.02.005 22376998

