# Supplementary material for: Temporal and regional trends of choking injuries in children in Italy, 2001–2013
Source: Inj Epidemiol. 2018 Aug 1;5:30. doi: 10.1186/s40621-018-0160-0 (PMC6068058; doi:10.1186/s40621-018-0160-0)
Supplement: Supplementary file 1 — Table S1. Hospitalization (95% Confidence Interval) Rate Ratio for male compared to female, estimated using a Negative Binomial. Table S2. Hospitalization (95% Confidence Interval) rate in male children (over 100,000 person-years). Aggregated data have been provided for reasons of data disclosure for some regions. Table S3. Hospitalization (95% Confidence Interval) rate in female children (over 100,000 person-years). Aggregated data have been provided for reasons of data disclosure for some regions. Table S4. Characteristics of hospitalization in male infants. Table S5. Characteristics of hospitalization in female infants. Table S6. Characteristics of hospitalization in male toddlers. Table S7. Characteristics of hospitalization in female toddlers. Table S8. Characteristics of hospitalization in male children (4–14 years). Table S9. Characteristics of hospitalization in female children (4–14 years)x`. (DOCX 73 kb) [file 40621_2018_160_MOESM1_ESM.docx]

Table S1. Hospitalization (95% Confidence Interval) Rate Ratio for male compared to female, estimated using a Negative Binomial.

| Region | Negative Binomial RR (Male vs Female) | P-value |
| --- | --- | --- |
| Abruzzo | 1 [0.13,7.67] | 0.99 |
| Basilicata | 1.24 [0.17,8.92] | 0.82 |
| Calabria | 1.4 [0.14,13.75] | 0.75 |
| Campania | 1.01 [0.14,7.57] | 0.99 |
| Emilia Romagna | 1.14 [0.2,6.61] | 0.87 |
| Friuli Venezia Giulia | 1.18 [0.25,5.46] | 0.83 |
| Lazio | 0.87 [0.12,6.35] | 0.89 |
| Liguria | 1.06 [0.2,5.65] | 0.95 |
| Lombardia | 0.98 [0.16,6.03] | 0.98 |
| Marche | 0.9 [0.08,9.52] | 0.92 |
| Molise | 1.91 [0.17,21.78] | 0.56 |
| Piemonte | 1.1 [0.22,5.58] | 0.91 |
| Puglia | 0.89 [0.08,9.68] | 0.91 |
| Sardegna | 1.21 [0.2,7.42] | 0.83 |
| Sicilia | 0.89 [0.05,15.32] | 0.93 |
| Toscana | 1.4 [0.31,6.32] | 0.65 |
| Trentino Alto Adige | 1.05 [0.25,4.53] | 0.94 |
| Umbria | 1.21 [0.15,9.87] | 0.84 |
| Valle d'Aosta | 2.82 [0.23,148.22] | 0.67 |
| Veneto | 1.17 [0.18,7.66] | 0.86 |

Table S2. Hospitalization (95% Confidence Interval) rate in male children (over 100,000 person-years). Aggregated data have been provided for reasons of data disclosure for some regions.

|  | **2001** | **2002** | **2003** | **2004** | **2005** | **2006** | **2007** | **2008** | **2009** | **2010** | **2011** | **2012** | **2013** |
| --- | --- | --- | --- | --- | --- | --- | --- | --- | --- | --- | --- | --- | --- |
| **Abruzzo-Molise-Marche-Umbria** | 9.08 (13) [5.95,13.74] | 9.85 (23) [6.57,14.66] | 9.03 (16) [5.92,13.67] | 9.73 (22) [6.49,14.48] | 11.13 (19) [7.64,16.11] | 9.22 (24) [6.1,13.84] | 8.47 (13) [5.5,12.93] | 5.84 (19) [3.46,9.72] | 4.34 (15) [2.35,7.81] | 5.77 (11) [3.42,9.6] | 5.39 (17) [3.13,9.12] | 4.4 (6) [2.38,7.92] | 3.66 (46) [1.86,6.97] |
| **Calabria-Basilicata-Campania** | 8.93 (24) [7,11.37] | 7.63 (26) [5.85,9.94] | 6.66 (24) [4.99,8.85] | 9.31 (26) [7.3,11.86] | 7.11 (30) [5.36,9.4] | 5.97 (25) [4.37,8.11] | 5.93 (23) [4.33,8.09] | 4.72 (16) [3.3,6.72] | 3.34 (12) [2.17,5.1] | 3.81 (16) [2.54,5.68] | 5.62 (15) [4.03,7.8] | 2.73 (12) [1.67,4.41] | 1.99 (10) [1.11,3.51] |
| **Emilia Romagna** | - | 10.88 (69) [7.25,16.19] | 8.91 (58) [5.72,13.73] | 8.28 (50) [5.26,12.89] | 3.81 (69) [1.94,7.26] | 7.43 (52) [4.67,11.71] | 8.72 (43) [5.71,13.19] | 7.08 (42) [4.44,11.15] | 4.81 (33) [2.74,8.29] | 5.69 (23) [3.42,9.32] | 4.6 (26) [2.62,7.93] | 4.66 (38) [2.65,8.04] | 5.58 (18) [3.36,9.14] |
| **Veneto-Friuli-Trentino** | - | 17.11 (107) [13.64,21.44] | 12.96 (83) [10,16.77] | 11.5 (83) [8.75,15.09] | 12.11 (82) [9.3,15.73] | 8.69 (75) [6.37,11.82] | 8.97 (76) [6.62,12.12] | 4.71 (53) [3.09,7.13] | 8.31 (55) [6.08,11.3] | 8.41 (44) [6.19,11.4] | 6.08 (52) [4.23,8.69] | 6.37 (39) [4.46,9.06] | 6.15 (15) [4.28,8.79] |
| **Lazio-Toscana** | 6.11 (79) [4.32,8.6] | 6.62 (61) [4.75,9.19] | 6.89 (55) [4.99,9.49] | 8.34 (59) [6.23,11.12] | 6.35 (43) [4.56,8.82] | 3.98 (45) [2.61,6.02] | 6.75 (24) [4.93,9.22] | 3.64 (43) [2.36,5.56] | 3.74 (44) [2.45,5.65] | 3.69 (32) [2.42,5.58] | 3.66 (33) [2.4,5.53] | 6.17 (32) [4.44,8.52] | 4.52 (35) [3.09,6.59] |
| **Piemonte-V.D'aosta-Liguria** | - | 3.65 (48) [2.03,6.43] | 6.38 (30) [4.14,9.74] | 4.37 (32) [2.59,7.28] | 5.9 (35) [3.79,9.09] | 5.04 (40) [3.12,8.03] | 6.3 (33) [4.13,9.54] | 3.36 (22) [1.87,5.92] | 4.84 (41) [3,7.71] | 3.78 (32) [2.2,6.4] | 2.75 (27) [1.45,5.09] | 4.33 (19) [2.61,7.09] | 1.52 (68) [0.62,3.48] |
| **Lombardia** | - | 7.5 (38) [5.55,10.1] | 7.68 (40) [5.73,10.28] | 4.7 (49) [3.23,6.8] | 4.9 (38) [3.41,7.01] | 5.26 (24) [3.72,7.41] | 5.91 (42) [4.28,8.14] | 4.79 (23) [3.35,6.81] | 3.13 (24) [2.01,4.83] | 5.73 (24) [4.17,7.86] | 4.41 (24) [3.06,6.3] | 3.79 (39) [2.55,5.6] | 2.63 (29) [1.63,4.2] |
| **Puglia** | 19.43 (65) [15.2,24.78] | 18.85 (45) [14.67,24.19] | 13.22 (45) [9.76,17.86] | 13.4 (39) [9.88,18.09] | 11.68 (49) [8.42,16.14] | 14.87 (47) [11.11,19.82] | 14.5 (32) [10.78,19.46] | 9.99 (25) [6.95,14.29] | 7.91 (31) [5.23,11.87] | 9.89 (23) [6.84,14.23] | 7.41 (18) [4.81,11.32] | 5.93 (24) [3.63,9.58] | 7.99 (41) [5.24,12.09] |
| **Islands (Sardegna and Sicilia)** | 7.3 (13) [5.3,10] | 19.41 (26) [15.98,23.55] | 15.23 (22) [12.2,18.98] | 15.37 (21) [12.32,19.16] | 15.37 (10) [12.3,19.19] | 14.25 (20) [11.29,17.97] | 14.66 (24) [11.63,18.45] | 10.36 (20) [7.84,13.66] | 10.85 (14) [8.25,14.23] | 8.74 (17) [6.42,11.84] | 10.4 (14) [7.85,13.75] | 8.04 (14) [5.8,11.11] | 3.12 (17) [1.81,5.27] |
| **Molise-Marche-Umbria** | 9.08 (13) [5.95,13.74] | 9.85 (23) [6.57,14.66] | 9.03 (16) [5.92,13.67] | 9.73 (22) [6.49,14.48] | 11.13 (19) [7.64,16.11] | 9.22 (24) [6.1,13.84] | 8.47 (13) [5.5,12.93] | 5.84 (19) [3.46,9.72] | 4.34 (15) [2.35,7.81] | 5.77 (11) [3.42,9.6] | 5.39 (17) [3.13,9.12] | 4.4 (6) [2.38,7.92] | 3.66 (46) [1.86,6.97] |

Table S3. Hospitalization (95% Confidence Interval) rate in female children (over 100,000 person-years). Aggregated data have been provided for reasons of data disclosure for some regions

|  | **2001** | **2002** | **2003** | **2004** | **2005** | **2006** | **2007** | **2008** | **2009** | **2010** | **2011** | **2012** | **2013** |
| --- | --- | --- | --- | --- | --- | --- | --- | --- | --- | --- | --- | --- | --- |
| **Abruzzo-Molise-Marche-Umbria** | 11.61 (12) [7.92,16.91] | 13.63 (20) [9.59,19.28] | 8.37 (15) [5.32,13.03] | 7.14 (14) [4.36,11.53] | 5.12 (7) [2.85,9] | 4.32 (11) [2.27,7.98] | 5.48 (8) [3.12,9.45] | 8.93 (7) [5.79,13.63] | 5 (11) [2.78,8.8] | 6.14 (19) [3.63,10.21] | 2.29 (8) [0.93,5.26] | 3.88 (11) [1.97,7.4] | 3.88 (45) [1.97,7.39] |
| **Calabria-Basilicata-Campania** | 8.16 (29) [6.28,10.58] | 5.53 (34) [4,7.61] | 5.32 (21) [3.82,7.38] | 3.97 (18) [2.69,5.82] | 5.6 (13) [4.04,7.74] | 4.66 (11) [3.24,6.67] | 5.19 (14) [3.67,7.3] | 4.66 (23) [3.22,6.71] | 2.44 (13) [1.44,4.06] | 5.39 (16) [3.81,7.58] | 4.82 (6) [3.33,6.94] | 2.56 (10) [1.52,4.26] | 0.97 (10) [0.39,2.23] |
| **Emilia Romagna** | - | 9.3 (60) [5.91,14.48] | 6.02 (40) [3.42,10.37] | 6.27 (38) [3.64,10.61] | 5.67 (28) [3.22,9.76] | 2.77 (39) [1.21,5.98] | 5.8 (32) [3.37,9.81] | 5.66 (35) [3.29,9.57] | 6.95 (31) [4.31,11.08] | 3.56 (16) [1.81,6.79] | 4.2 (35) [2.27,7.56] | 4.58 (31) [2.55,8.06] | 3.13 (16) [1.53,6.18] |
| **Veneto-Friuli-Trentino** | - | 10.28 (88) [7.59,13.88] | 13.69 (74) [10.56,17.71] | 12.6 (86) [9.63,16.45] | 6.51 (71) [4.47,9.43] | 8.35 (51) [6.02,11.53] | 6.55 (69) [4.53,9.42] | 5.83 (69) [3.95,8.55] | 6.56 (63) [4.56,9.38] | 5.07 (49) [3.35,7.61] | 3.22 (33) [1.91,5.36] | 3.66 (42) [2.24,5.91] | 3.25 (26) [1.92,5.4] |
| **Lazio-Toscana** | 5.71 (45) [3.95,8.21] | 6.8 (61) [4.86,9.48] | 4.91 (57) [3.3,7.25] | 5.74 (30) [3.99,8.2] | 6 (39) [4.22,8.48] | 5.07 (31) [3.46,7.38] | 6.31 (28) [4.5,8.79] | 3.53 (32) [2.24,5.49] | 2.47 (25) [1.44,4.18] | 2.93 (16) [1.79,4.74] | 3.22 (18) [2.02,5.08] | 2.67 (16) [1.58,4.44] | 3.8 (31) [2.47,5.8] |
| **Piemonte-V.D'aosta-Liguria** | - | 3.56 (39) [1.93,6.42] | 5.87 (41) [3.68,9.24] | 4.34 (32) [2.52,7.34] | 3.97 (31) [2.26,6.85] | 1.96 (28) [0.86,4.24] | 3.06 (20) [1.61,5.65] | 2.19 (32) [1.02,4.5] | 1.89 (18) [0.83,4.08] | 2.93 (14) [1.54,5.42] | 5.03 (21) [3.12,8.02] | 2.15 (13) [1,4.42] | 2.94 (44) [1.55,5.44] |
| **Lombardia** | - | 7.76 (37) [5.73,10.49] | 6.61 (27) [4.76,9.13] | 6.82 (32) [4.96,9.34] | 5.2 (34) [3.61,7.43] | 4.94 (29) [3.42,7.11] | 4.39 (37) [2.97,6.44] | 3.08 (21) [1.93,4.85] | 4.83 (15) [3.36,6.91] | 2.67 (18) [1.63,4.31] | 2.05 (20) [1.16,3.53] | 3.12 (16) [1.98,4.85] | 1.91 (23) [1.06,3.36] |
| **Puglia** | 13.34 (65) [9.81,18.07] | 19.97 (47) [15.54,25.62] | 14.63 (42) [10.87,19.63] | 13.24 (44) [9.66,18.08] | 13.95 (62) [10.26,18.9] | 19.92 (40) [15.4,25.71] | 13.05 (28) [9.45,17.96] | 9.24 (28) [6.26,13.55] | 9.36 (32) [6.34,13.72] | 10.78 (24) [7.5,15.41] | 8.15 (14) [5.34,12.33] | 4.86 (10) [2.76,8.37] | 3.51 (28) [1.78,6.68] |
| **Islands (Sardegna and Sicilia)** | 5.24 (6) [3.55,7.69] | 16.81 (21) [13.56,20.81] | 14.3 (14) [11.31,18.06] | 16.81 (15) [13.52,20.86] | 14.06 (14) [11.06,17.84] | 10.24 (7) [7.7,13.57] | 14.06 (15) [11.02,17.91] | 14.27 (15) [11.18,18.17] | 13.17 (19) [10.2,16.97] | 10.32 (10) [7.72,13.76] | 7 (12) [4.89,9.95] | 9.13 (13) [6.66,12.46] | 5.69 (9) [3.8,8.47] |
| **Molise-Marche-Umbria** | 11.61 (12) [7.92,16.91] | 13.63 (20) [9.59,19.28] | 8.37 (15) [5.32,13.03] | 7.14 (14) [4.36,11.53] | 5.12 (7) [2.85,9] | 4.32 (11) [2.27,7.98] | 5.48 (8) [3.12,9.45] | 8.93 (7) [5.79,13.63] | 5 (11) [2.78,8.8] | 6.14 (19) [3.63,10.21] | 2.29 (8) [0.93,5.26] | 3.88 (11) [1.97,7.4] | 3.88 (45) [1.97,7.39] |

Table S4. Characteristics of hospitalization in male infants

|  | **2001** | **2002** | **2003** | **2004** | **2005** | **2006** | **2007** | **2008** | **2009** | **2010** | **2011** | **2012** | **2013** |
| --- | --- | --- | --- | --- | --- | --- | --- | --- | --- | --- | --- | --- | --- |
|  | **(N=128)** | **(N=237)** | **(N=214)** | **(N=212)** | **(N=185)** | **(N=160)** | **(N=137)** | **(N=113)** | **(N=101)** | **(N=96)** | **(N=115)** | **(N=75)** | **(N=47)** |
| **Age** *(days)* | 43.00 [14.00 132.00] | 35.00 [13.00; 73.00] | 37.50 [13.25; 111.75] | 44.50 [17.00; 124.25] | 43.00 [16.00; 98.00] | 43.00/[16.00; 107.75] | 44.00 [17.00; 112.00] | 52.00/ [17.00; 128.00] | 46.00 [20.00; 146.00] | 45.50 [16-75; 110.50] | 56.00 [17.00; 158.50] | 56.00 [17.50; 265.50] | 74.00 [41.50; 234.00] |
| **Hospitalization:** *Day hospital service* | 0% ( 0) | 0% ( 1) | 1% ( 3) | 2% ( 5) | 2% ( 3) | 0% ( 0) | 1% ( 1) | 2% ( 2) | 0% ( 0) | 0% ( 0) | 0% ( 0) | 1% ( 1) | 0% ( 0) |
| **Length of stay** (only for regular inpatient hospitalization) *(days)* | 2.0 [1.0; 3.0] | 2.0 [1.0; 3.0] | 2.0 [1.0; 3.0] | 2.0 [1.0; 3.0] | 2.0 [1.0; 3.0] | 2.0 [1.0; 3.0] | 2.0 [1.0; 3.0] | 2.0 [1.0; 3.0] | 2.0 [1.0; 3.0] | 2.0 [1.0; 3.0] | 2.0 [1.0; 3.0] | 2.0 [1.0; 3.0] | 2.0 [1.0; 3.5] |
| **Discharge modalities:** *Exitus* | 0% ( 0) | 0% ( 0) | 0% ( 0) | 0% ( 1) | 1% ( 2) | 0% ( 0) | 0% ( 0) | 0% ( 0) | 0% ( 0) | 0% ( 0) | 0% ( 0) | 0% ( 0) | 2% ( 1) |
| *Home* | 83% ( 106) | 83% ( 197) | 87% ( 186) | 85% ( 180) | 85% ( 157) | 84% ( 135) | 83% ( 114) | 89% ( 101) | 86% ( 87) | 85% ( 82) | 82% ( 94) | 81% ( 61) | 77% ( 36) |
| *Nursing home* | 0% ( 0) | 0% ( 0) | 0% ( 0) | 0% ( 0) | 0% ( 0) | 0% ( 0) | 0% ( 0) | 0% ( 0) | 0% ( 0) | 0% ( 0) | 0% ( 0) | 0% ( 0) | 0% ( 0) |
| *Home hospitalization* | 2% ( 2) | 0% ( 0) | 0% ( 1) | 1% ( 3) | 1% ( 2) | 0% ( 0) | 0% ( 0) | 0% ( 0) | 0% ( 0) | 0% ( 0) | 0% ( 0) | 0% ( 0) | 0% ( 0) |
| *Discharge against medical advice* | 16% ( 20) | 16% ( 37) | 12% ( 25) | 11% ( 23) | 12% ( 22) | 13% ( 21) | 14% ( 19) | 11% ( 12) | 14% ( 14) | 11% ( 11) | 16% ( 18) | 13% ( 10) | 19% ( 9) |
| *Transfer to another hospital* | 0% ( 0) | 1% ( 3) | 1% ( 2) | 2% ( 5) | 1% ( 2) | 2% ( 4) | 3% ( 4) | 0% ( 0) | 0% ( 0) | 3% ( 3) | 3% ( 3) | 5% ( 4) | 2% ( 1) |
| *Transfer in the same hospital* | 0% ( 0) | 0% ( 0) | 0% ( 0) | 0% ( 0) | 0% ( 0) | 0% ( 0) | 0% ( 0) | 0% ( 0) | 0% ( 0) | 0% ( 0) | 0% ( 0) | 0% ( 0) | 0% ( 0) |
| *Rehabilitation* | 0% ( 0) | 0% ( 0) | 0% ( 0) | 0% ( 0) | 0% ( 0) | 0% ( 0) | 0% ( 0) | 0% ( 0) | 0% ( 0) | 0% ( 0) | 0% ( 0) | 0% ( 0) | 0% ( 0) |
| *Home care assistance* | 0% ( 0) | 0% ( 0) | 0% ( 0) | 0% ( 0) | 0% ( 0) | 0% ( 0) | 0% ( 0) | 0% ( 0) | 0% ( 0) | 0% ( 0) | 0% ( 0) | 0% ( 0) | 0% ( 0) |
| **Hospitalization type:** *Newborn* | 0% ( 0) | 0% ( 0) | 0% ( 0) | 0% ( 0) | 1% ( 1) | 0% ( 0) | 0% ( 0) | 0% ( 0) | 0% ( 0) | 0% ( 0) | 0% ( 0) | 0% ( 0) | 0% ( 0) |
| *Non-urgent hospitalization* | 21% ( 27) | 15% ( 35) | 15% ( 32) | 6% ( 13) | 7% ( 12) | 4% ( 6) | 8% ( 11) | 4% ( 4) | 4% ( 4) | 6% ( 6) | 3% ( 3) | 3% ( 2) | 0% ( 0) |
| *Urgent hospitalization* | 79% ( 100) | 85% ( 197) | 84% ( 180) | 93% ( 192) | 92% ( 169) | 96% ( 151) | 91% ( 124) | 96% ( 107) | 96% ( 97) | 94% ( 88) | 97% ( 109) | 97% ( 70) | 100% ( 46) |
| *Planned hospitalization* | 0% ( 0) | 0% ( 0) | 0% ( 1) | 0% ( 0) | 1% ( 1) | 0% ( 0) | 0% ( 0) | 0% ( 0) | 0% ( 0) | 0% ( 0) | 0% ( 0) | 0% ( 0) | 0% ( 0) |
| *Other* | 0% ( 0) | 0% ( 0) | 0% ( 1) | 0% ( 1) | 0% ( 0) | 0% ( 0) | 1% ( 1) | 0% ( 0) | 0% ( 0) | 0% ( 0) | 0% ( 0) | 0% ( 0) | 0% ( 0) |
| **Trauma:** *Other* | 97% ( 32) | 92% ( 70) | 85% ( 51) | 89% ( 70) | 91% ( 58) | 91% ( 53) | 69% ( 33) | 90% ( 26) | 94% ( 33) | 89% ( 33) | 77% ( 40) | 75% ( 12) | 65% ( 13) |
| *Work-related injury* | 0% ( 0) | 0% ( 0) | 0% ( 0) | 0% ( 0) | 0% ( 0) | 0% ( 0) | 2% ( 1) | 0% ( 0) | 0% ( 0) | 0% ( 0) | 0% ( 0) | 6% ( 1) | 0% ( 0) |
| *Home accident* | 3% ( 1) | 8% ( 6) | 15% ( 9) | 10% ( 8) | 9% ( 6) | 9% ( 5) | 29% ( 14) | 10% ( 3) | 6% ( 2) | 11% ( 4) | 21% ( 11) | 19% ( 3) | 35% ( 7) |
| *Car accident* | 0% ( 0) | 0% ( 0) | 0% ( 0) | 0% ( 0) | 0% ( 0) | 0% ( 0) | 0% ( 0) | 0% ( 0) | 0% ( 0) | 0% ( 0) | 0% ( 0) | 0% ( 0) | 0% ( 0) |
| *Violence* | 0% ( 0) | 0% ( 0) | 0% ( 0) | 0% ( 0) | 0% ( 0) | 0% ( 0) | 0% ( 0) | 0% ( 0) | 0% ( 0) | 0% ( 0) | 0% ( 0) | 0% ( 0) | 0% ( 0) |
| *Self-inflicted injury* | 0% ( 0) | 0% ( 0) | 0% ( 0) | 1% ( 1) | 0% ( 0) | 0% ( 0) | 0% ( 0) | 0% ( 0) | 0% ( 0) | 0% ( 0) | 2% ( 1) | 0% ( 0) | 0% ( 0) |
| **DRG type:** medical | 100% ( 128) | 100% ( 236) | 100% ( 213) | 100% ( 212) | 100% ( 185) | 99% ( 159) | 100% ( 137) | 100% ( 113) | 100% ( 101) | 100% ( 96) | 99% ( 114) | 99% ( 74) | 100% ( 47) |
| **Nationality:** *Italian* | 100% ( 128) | 96% ( 227) | 97% ( 208) | 97% ( 205) | 95% ( 176) | 96% ( 153) | 96% ( 132) | 94% ( 106) | 94% ( 95) | 96% ( 92) | 90% ( 104) | 85% ( 64) | 85% ( 40) |
| *Albanian* | 0% ( 0) | 1% ( 3) | 0% ( 0) | 2% ( 4) | 0% ( 0) | 1% ( 2) | 1% ( 1) | 0% ( 0) | 0% ( 0) | 0% ( 0) | 1% ( 1) | 1% ( 1) | 2% ( 1) |
| *Romanian* | 0% ( 0) | 0% ( 1) | 0% ( 0) | 0% ( 0) | 2% ( 3) | 0% ( 0) | 1% ( 2) | 1% ( 1) | 1% ( 1) | 0% ( 0) | 3% ( 3) | 4% ( 3) | 2% ( 1) |
| *Moroccan* | 0% ( 0) | 1% ( 2) | 0% ( 1) | 0% ( 1) | 1% ( 2) | 2% ( 3) | 1% ( 1) | 3% ( 3) | 3% ( 3) | 1% ( 1) | 2% ( 2) | 0% ( 0) | 2% ( 1) |
| *Tunisian* | 0% ( 0) | 0% ( 0) | 1% ( 2) | 0% ( 0) | 0% ( 0) | 0% ( 0) | 0% ( 0) | 0% ( 0) | 1% ( 1) | 2% ( 2) | 1% ( 1) | 0% ( 0) | 2% ( 1) |
| **Main procedure:** |  |  |  |  |  |  |  |  |  |  |  |  |  |
| *Fiber-optic bronchoscopy* | 0% ( 0) | 0% ( 0) | 0% ( 0) | 4% ( 4) | 1% ( 1) | 2% ( 1) | 2% ( 1) | 8% ( 5) | 6% ( 3) | 3% ( 1) | 2% ( 1) | 5% ( 2) | 5% ( 1) |
| *Routine chest x-ray* | 16% ( 3) | 12% ( 9) | 16% ( 13) | 16% ( 16) | 14% ( 10) | 11% ( 6) | 23% ( 11) | 11% ( 7) | 10% ( 5) | 16% ( 6) | 15% ( 8) | 14% ( 6) | 0% ( 0) |
| *Diagnostic ultrasound of head and neck* | 5% ( 1) | 4% ( 3) | 6% ( 5) | 6% ( 6) | 3% ( 2) | 7% ( 4) | 8% ( 4) | 6% ( 4) | 4% ( 2) | 3% ( 1) | 4% ( 2) | 2% ( 1) | 0% ( 0) |
| *Diagnostic ultrasound of heart* | 5% ( 1) | 1% ( 1) | 2% ( 2) | 8% ( 8) | 1% ( 1) | 2% ( 1) | 0% ( 0) | 5% ( 3) | 2% ( 1) | 0% ( 0) | 2% ( 1) | 7% ( 3) | 14% ( 3) |
| *Diagnostic interview and evaluation, not otherwise specified* | 5% ( 1) | 3% ( 2) | 1% ( 1) | 5% ( 5) | 7% ( 5) | 7% ( 4) | 4% ( 2) | 0% ( 0) | 0% ( 0) | 0% ( 0) | 2% ( 1) | 5% ( 2) | 0% ( 0) |
| *Electrocardiogram* | 11% ( 2) | 17% ( 13) | 13% ( 11) | 18% ( 18) | 13% ( 9) | 12% ( 7) | 21% ( 10) | 14% ( 9) | 20% ( 10) | 8% ( 3) | 2% ( 1) | 9% ( 4) | 0% ( 0) |
| *General physical examination* | 0% ( 0) | 3% ( 2) | 2% ( 2) | 2% ( 2) | 3% ( 2) | 4% ( 2) | 2% ( 1) | 3% ( 2) | 6% ( 3) | 14% ( 5) | 6% ( 3) | 9% ( 4) | 10% ( 2) |
| *Microscopic examination of blood, other microscopic examination* | 16% ( 3) | 14% ( 11) | 10% ( 8) | 7% ( 7) | 10% ( 7) | 20% ( 11) | 12% ( 6) | 14% ( 9) | 12% ( 6) | 19% ( 7) | 15% ( 8) | 16% ( 7) | 19% ( 4) |
| **Secondary procedure (1):** |  |  |  |  |  |  |  |  |  |  |  |  |  |
| *Routine chest x-ray* | 21% ( 13) | 17% ( 16) | 13% ( 11) | 6% ( 5) | 9% ( 8) | 9% ( 7) | 12% ( 7) | 11% ( 5) | 8% ( 4) | 7% ( 4) | 5% ( 3) | 16% ( 6) | 6% ( 2) |
| *Diagnostic ultrasound of head and neck* | 8% ( 5) | 5% ( 5) | 4% ( 3) | 7% ( 6) | 8% ( 7) | 1% ( 1) | 2% ( 1) | 0% ( 0) | 4% ( 2) | 11% ( 6) | 0% ( 0) | 0% ( 0) | 0% ( 0) |
| *Interview and evaluation, described as comprehensive* | 15% ( 9) | 4% ( 4) | 5% ( 4) | 6% ( 5) | 6% ( 5) | 3% ( 2) | 5% ( 3) | 0% ( 0) | 0% ( 0) | 4% ( 2) | 0% ( 0) | 3% ( 1) | 0% ( 0) |
| *Electroencephalogram* | 8% ( 5) | 4% ( 4) | 4% ( 3) | 5% ( 4) | 2% ( 2) | 5% ( 4) | 5% ( 3) | 2% ( 1) | 0% ( 0) | 0% ( 0) | 2% ( 1) | 3% ( 1) | 3% ( 1) |
| *Electrocardiogram* | 8% ( 5) | 20% ( 18) | 16% ( 13) | 16% ( 13) | 17% ( 15) | 14% ( 10) | 14% ( 8) | 13% ( 6) | 16% ( 8) | 16% ( 9) | 30% ( 17) | 13% ( 5) | 22% ( 7) |
| *General physical examination* | 2% ( 1) | 5% ( 5) | 5% ( 4) | 7% ( 6) | 2% ( 2) | 7% ( 5) | 7% ( 4) | 4% ( 2) | 4% ( 2) | 4% ( 2) | 4% ( 2) | 5% ( 2) | 0% ( 0) |
| *Microscopic examination of blood, other microscopic examination* | 5% ( 3) | 8% ( 7) | 7% ( 6) | 7% ( 6) | 7% ( 6) | 19% ( 14) | 5% ( 3) | 9% ( 4) | 14% ( 7) | 12% ( 7) | 16% ( 9) | 16% ( 6) | 22% ( 7) |
| *Other oxygen enrichment* | 0% ( 0) | 5% ( 5) | 6% ( 5) | 4% ( 3) | 5% ( 4) | 4% ( 3) | 5% ( 3) | 2% ( 1) | 2% ( 1) | 5% ( 3) | 0% ( 0) | 0% ( 0) | 0% ( 0) |

Table S5. Characteristics of hospitalization in female infants

|  | **2001** | **2002** | **2003** | **2004** | **2005** | **2006** | **2007** | **2008** | **2009** | **2010** | **2011** | **2012** | **2013** |
| --- | --- | --- | --- | --- | --- | --- | --- | --- | --- | --- | --- | --- | --- |
|  | **(N=128)** | **(N=249)** | **(N=226)** | **(N=219)** | **(N=183)** | **(N=160)** | **(N=147)** | **(N=152)** | **(N=116)** | **(N=111)** | **(N=101)** | **(N=79)** | **(N=53)** |
| **Age** *(days)* | 35.50 [14.00; 86.25] | 31.00 [15.00; 79.00] | 35.00 [14.00; 84.00] | 33.00/ [13.00; 71.50] | 42.00 [14.50; 86.00] | 37.00 [16.00; 99.00] | 40.00 [14.00; 90.00] | 38.50 [1]3.00; 91.50] | 46.00 [15.00; 101.50] | 46.00 [14.50; 130.00] | 35.00 [16.00; 66.00] | 52.00 [22.50; 144.00] | 42.00 [19.00; 73.00] |
| **Hospitalization:** *Day hospital service* | 2% ( 2) | 1% ( 2) | 1% ( 3) | 0% ( 1) | 0% ( 0) | 0% ( 0) | 0% ( 0) | 0% ( 0) | 1% ( 1) | 1% ( 1) | 0% ( 0) | 0% ( 0) | 0% ( 0) |
| **Length of stay** (only for regular inpatient hospitalization**)** *(days)* | 2 [1; 3] | 2 [1; 3] | 2 [1; 3] | 2 [1; 3] | 2 [1; 3] | 2 [1; 3] | 2 [1; 3] | 2 [1; 3] | 2 [1; 2] | 2 [1; 3] | 2 [1; 3] | 2 [1; 3] | 2 [2; 3] |
| **Discharge modalities:** *Exitus* | 0% ( 0) | 0% ( 0) | 0% ( 0) | 0% ( 0) | 0% ( 0) | 0% ( 0) | 0% ( 0) | 0% ( 0) | 0% ( 0) | 0% ( 0) | 1% ( 1) | 0% ( 0) | 0% ( 0) |
| *Home* | 80% ( 102) | 84% ( 210) | 84% ( 190) | 84% ( 184) | 86% ( 157) | 82% ( 131) | 90% ( 132) | 78% ( 119) | 82% ( 95) | 86% ( 95) | 82% ( 83) | 87% ( 69) | 85% ( 45) |
| *Nursing home* | 0% ( 0) | 0% ( 0) | 0% ( 0) | 0% ( 0) | 0% ( 0) | 0% ( 0) | 0% ( 0) | 0% ( 0) | 0% ( 0) | 0% ( 0) | 0% ( 0) | 0% ( 0) | 0% ( 0) |
| *Home hospitalization* | 2% ( 3) | 1% ( 2) | 0% ( 1) | 0% ( 1) | 1% ( 1) | 0% ( 0) | 1% ( 1) | 1% ( 2) | 0% ( 0) | 0% ( 0) | 0% ( 0) | 0% ( 0) | 0% ( 0) |
| *Discharge against medical advice* | 18% ( 23) | 13% ( 33) | 15% ( 33) | 13% ( 28) | 13% ( 23) | 16% ( 25) | 9% ( 13) | 19% ( 29) | 17% ( 20) | 13% ( 14) | 17% ( 17) | 13% ( 10) | 13% ( 7) |
| *Transfer to another hospital* | 0% ( 0) | 2% ( 4) | 1% ( 2) | 2% ( 5) | 1% ( 2) | 2% ( 4) | 1% ( 1) | 1% ( 1) | 1% ( 1) | 2% ( 2) | 0% ( 0) | 0% ( 0) | 2% ( 1) |
| *Transfer in the same hospital* | 0% ( 0) | 0% ( 0) | 0% ( 0) | 0% ( 1) | 0% ( 0) | 0% ( 0) | 0% ( 0) | 0% ( 0) | 0% ( 0) | 0% ( 0) | 0% ( 0) | 0% ( 0) | 0% ( 0) |
| *Rehabilitation* | 0% ( 0) | 0% ( 0) | 0% ( 0) | 0% ( 0) | 0% ( 0) | 0% ( 0) | 0% ( 0) | 1% ( 1) | 0% ( 0) | 0% ( 0) | 0% ( 0) | 0% ( 0) | 0% ( 0) |
| *Home care assistance* | 0% ( 0) | 0% ( 0) | 0% ( 0) | 0% ( 0) | 0% ( 0) | 0% ( 0) | 0% ( 0) | 0% ( 0) | 0% ( 0) | 0% ( 0) | 0% ( 0) | 0% ( 0) | 0% ( 0) |
| **Hospitalization type:** Newborn | 0% ( 0) | 0% ( 0) | 0% ( 0) | 0% ( 0) | 0% ( 0) | 0% ( 0) | 0% ( 0) | 0% ( 0) | 0% ( 0) | 0% ( 0) | 0% ( 0) | 0% ( 0) | 0% ( 0) |
| *Non-urgent hospitalization* | 15% ( 19) | 16% ( 39) | 9% ( 20) | 6% ( 12) | 7% ( 12) | 3% ( 5) | 10% ( 14) | 5% ( 8) | 4% ( 4) | 5% ( 5) | 3% ( 3) | 3% ( 2) | 2% ( 1) |
| *Urgent hospitalization* | 84% ( 107) | 84% ( 210) | 89% ( 201) | 93% ( 202) | 93% ( 171) | 97% ( 154) | 90% ( 132) | 95% ( 144) | 96% ( 110) | 95% ( 105) | 97% ( 97) | 97% ( 77) | 98% ( 51) |
| *Planned hospitalization* | 1% ( 1) | 0% ( 0) | 1% ( 2) | 0% ( 0) | 0% ( 0) | 0% ( 0) | 0% ( 0) | 0% ( 0) | 0% ( 0) | 0% ( 0) | 0% ( 0) | 0% ( 0) | 0% ( 0) |
| *Other* | 0% ( 0) | 0% ( 0) | 1% ( 3) | 2% ( 4) | 0% ( 0) | 0% ( 0) | 0% ( 0) | 0% ( 0) | 0% ( 0) | 0% ( 0) | 0% ( 0) | 0% ( 0) | 0% ( 0) |
| **Trauma**: *Other* | 100% ( 27) | 90% ( 70) | 85% ( 68) | 93% ( 71) | 89% ( 57) | 83% ( 38) | 82% ( 41) | 87% ( 46) | 94% ( 30) | 87% ( 39) | 82% ( 27) | 72% ( 13) | 85% ( 17) |
| *Work-related injury* | 0% ( 0) | 0% ( 0) | 0% ( 0) | 0% ( 0) | 0% ( 0) | 0% ( 0) | 0% ( 0) | 0% ( 0) | 0% ( 0) | 0% ( 0) | 0% ( 0) | 0% ( 0) | 0% ( 0) |
| *Home accident* | 0% ( 0) | 10% ( 8) | 12% ( 10) | 7% ( 5) | 11% ( 7) | 17% ( 8) | 18% ( 9) | 13% ( 7) | 6% ( 2) | 13% ( 6) | 18% ( 6) | 28% ( 5) | 15% ( 3) |
| *Car accident* | 0% ( 0) | 0% ( 0) | 0% ( 0) | 0% ( 0) | 0% ( 0) | 0% ( 0) | 0% ( 0) | 0% ( 0) | 0% ( 0) | 0% ( 0) | 0% ( 0) | 0% ( 0) | 0% ( 0) |
| *Violence* | 0% ( 0) | 0% ( 0) | 0% ( 0) | 0% ( 0) | 0% ( 0) | 0% ( 0) | 0% ( 0) | 0% ( 0) | 0% ( 0) | 0% ( 0) | 0% ( 0) | 0% ( 0) | 0% ( 0) |
| *Self-inflicted injury* | 0% ( 0) | 0% ( 0) | 2% ( 2) | 0% ( 0) | 0% ( 0) | 0% ( 0) | 0% ( 0) | 0% ( 0) | 0% ( 0) | 0% ( 0) | 0% ( 0) | 0% ( 0) | 0% ( 0) |
| **DRG type:** *medical* | 100% ( 128) | 100% ( 249) | 100% ( 226) | 100% ( 219) | 100% ( 183) | 100% ( 160) | 100% ( 147) | 99% ( 151) | 99% ( 115) | 100% ( 111) | 100% ( 101) | 100% ( 79) | 100% ( 53) |
| **Nationality :** *Italian* | 99% ( 126) | 98% ( 243) | 97% ( 219) | 96% ( 209) | 96% ( 176) | 96% ( 153) | 95% ( 140) | 96% ( 146) | 91% ( 105) | 94% ( 104) | 92% ( 93) | 91% ( 72) | 91% ( 48) |
| *Albanian* | 0% ( 0) | 0% ( 1) | 1% ( 2) | 1% ( 3) | 1% ( 2) | 1% ( 1) | 1% ( 2) | 2% ( 3) | 3% ( 3) | 2% ( 2) | 1% ( 1) | 4% ( 3) | 2% ( 1) |
| *Romanian* | 0% ( 0) | 0% ( 1) | 0% ( 0) | 0% ( 0) | 1% ( 1) | 1% ( 1) | 0% ( 0) | 1% ( 1) | 1% ( 1) | 1% ( 1) | 0% ( 0) | 1% ( 1) | 0% ( 0) |
| *Moroccan* | 0% ( 0) | 1% ( 2) | 0% ( 1) | 0% ( 0) | 2% ( 3) | 1% ( 1) | 2% ( 3) | 0% ( 0) | 2% ( 2) | 1% ( 1) | 4% ( 4) | 1% ( 1) | 4% ( 2) |
| *Tunisian* | 0% ( 0) | 0% ( 0) | 0% ( 1) | 1% ( 2) | 0% ( 0) | 0% ( 0) | 0% ( 0) | 0% ( 0) | 1% ( 1) | 0% ( 0) | 1% ( 1) | 0% ( 0) | 2% ( 1) |
| **Main procedure:** |  |  |  |  |  |  |  |  |  |  |  |  |  |
| *Routine chest x-ray* | 27% ( 6) | 13% ( 11) | 16% ( 13) | 18% ( 15) | 8% ( 5) | 9% ( 5) | 15% ( 9) | 18% ( 11) | 7% ( 4) | 2% ( 1) | 2% ( 1) | 6% ( 3) | 8% ( 3) |
| *Other chest x-ray* | 5% ( 1) | 5% ( 4) | 2% ( 2) | 5% ( 4) | 0% ( 0) | 0% ( 0) | 3% ( 2) | 2% ( 1) | 4% ( 2) | 6% ( 3) | 2% ( 1) | 2% ( 1) | 0% ( 0) |
| *Diagnostic ultrasound of head and neck* | 5% ( 1) | 5% ( 4) | 4% ( 3) | 7% ( 6) | 5% ( 3) | 4% ( 2) | 5% ( 3) | 2% ( 1) | 7% ( 4) | 6% ( 3) | 5% ( 2) | 2% ( 1) | 5% ( 2) |
| *Diagnostic ultrasound of heart* | 9% ( 2) | 4% ( 3) | 5% ( 4) | 2% ( 2) | 12% ( 7) | 5% ( 3) | 5% ( 3) | 3% ( 2) | 2% ( 1) | 4% ( 2) | 0% ( 0) | 0% ( 0) | 11% ( 4) |
| *Electroencephalogram* | 9% ( 2) | 6% ( 5) | 7% ( 6) | 5% ( 4) | 3% ( 2) | 5% ( 3) | 2% ( 1) | 7% ( 4) | 4% ( 2) | 4% ( 2) | 5% ( 2) | 4% ( 2) | 8% ( 3) |
| *Electrocardiogram* | 5% ( 1) | 18% ( 15) | 20% ( 16) | 15% ( 13) | 15% ( 9) | 16% ( 9) | 14% ( 8) | 10% ( 6) | 11% ( 6) | 14% ( 7) | 12% ( 5) | 10% ( 5) | 13% ( 5) |
| *General physical examination* | 5% ( 1) | 5% ( 4) | 2% ( 2) | 6% ( 5) | 0% ( 0) | 2% ( 1) | 0% ( 0) | 3% ( 2) | 7% ( 4) | 2% ( 1) | 10% ( 4) | 17% ( 8) | 11% ( 4) |
| *Microscopic examination of blood, other microscopic examination* | 5% ( 1) | 11% ( 9) | 10% ( 8) | 12% ( 10) | 22% ( 13) | 16% ( 9) | 12% ( 7) | 5% ( 3) | 21% ( 12) | 20% ( 10) | 28% ( 11) | 15% ( 7) | 21% ( 8) |
| **Secondary procedure (1):** |  |  |  |  |  |  |  |  |  |  |  |  |  |
| Routine chest x-ray | 22% ( 14) | 16% ( 18) | 15% ( 14) | 2% ( 2) | 14% ( 10) | 14% ( 10) | 8% ( 5) | 14% ( 10) | 12% ( 7) | 15% ( 8) | 5% ( 3) | 6% ( 3) | 3% ( 1) |
| Other chest x-ray | 5% ( 3) | 6% ( 7) | 6% ( 6) | 5% ( 5) | 5% ( 4) | 1% ( 1) | 3% ( 2) | 7% ( 5) | 0% ( 0) | 0% ( 0) | 0% ( 0) | 0% ( 0) | 3% ( 1) |
| Diagnostic ultrasound of heart | 6% ( 4) | 2% ( 2) | 2% ( 2) | 7% ( 7) | 5% ( 4) | 3% ( 2) | 3% ( 2) | 1% ( 1) | 4% ( 2) | 10% ( 5) | 5% ( 3) | 4% ( 2) | 0% ( 0) |
| Interview and evaluation, described as comprehensive | 13% ( 8) | 10% ( 11) | 4% ( 4) | 7% ( 7) | 4% ( 3) | 1% ( 1) | 5% ( 3) | 4% ( 3) | 4% ( 2) | 4% ( 2) | 9% ( 5) | 0% ( 0) | 0% ( 0) |
| Electroencephalogram | 5% ( 3) | 1% ( 1) | 5% ( 5) | 6% ( 6) | 0% ( 0) | 1% ( 1) | 3% ( 2) | 4% ( 3) | 2% ( 1) | 2% ( 1) | 2% ( 1) | 2% ( 1) | 3% ( 1) |
| Electrocardiogram | 24% ( 15) | 23% ( 25) | 21% ( 20) | 19% ( 20) | 19% ( 14) | 17% ( 12) | 23% ( 14) | 12% ( 9) | 21% ( 12) | 12% ( 6) | 13% ( 7) | 27% ( 13) | 29% ( 10) |
| General physical examination | 0% ( 0) | 4% ( 4) | 3% ( 3) | 7% ( 7) | 5% ( 4) | 10% ( 7) | 5% ( 3) | 0% ( 0) | 4% ( 2) | 8% ( 4) | 4% ( 2) | 2% ( 1) | 0% ( 0) |
| Microscopic examination of blood, other microscopic examination | 6% ( 4) | 5% ( 5) | 1% ( 1) | 9% ( 9) | 9% ( 7) | 11% ( 8) | 10% ( 6) | 11% ( 8) | 12% ( 7) | 10% ( 5) | 16% ( 9) | 18% ( 9) | 11% ( 4) |

Table S6. Characteristics of hospitalization in male toddlers

|  | **2001** | **2002** | **2003** | **2004** | **2005** | **2006** | **2007** | **2008** | **2009** | **2010** | **2011** | **2012** | **2013** |
| --- | --- | --- | --- | --- | --- | --- | --- | --- | --- | --- | --- | --- | --- |
|  | **(N=74)** | **(N=143)** | **(N=130)** | **(N=126)** | **(N=125)** | **(N=134)** | **(N=156)** | **(N=99)** | **(N=94)** | **(N=123)** | **(N=70)** | **(N=97)** | **(N=85)** |
| **Age** *(years)*: 1 | 26% ( 19) | 31% ( 45) | 35% ( 46) | 52% ( 66) | 59% ( 74) | 56% ( 75) | 46% ( 71) | 61% ( 60) | 62% ( 58) | 63% ( 77) | 54% ( 38) | 56% ( 54) | 53% ( 45) |
| 2 | 59% ( 44) | 47% ( 67) | 39% ( 51) | 30% ( 38) | 30% ( 38) | 35% ( 47) | 44% ( 69) | 28% ( 28) | 27% ( 25) | 26% ( 32) | 31% ( 22) | 32% ( 31) | 38% ( 32) |
| 3 | 15% ( 11) | 22% ( 31) | 25% ( 33) | 17% ( 22) | 10% ( 13) | 9% ( 12) | 10% ( 16) | 11% ( 11) | 12% ( 11) | 11% ( 14) | 14% ( 10) | 12% ( 12) | 9% ( 8) |
| **Hospitalization:** *Day hospital service* | 1% ( 1) | 1% ( 1) | 4% ( 5) | 3% ( 4) | 1% ( 1) | 1% ( 2) | 1% ( 1) | 4% ( 4) | 4% ( 4) | 1% ( 1) | 1% ( 1) | 1% ( 1) | 4% ( 3) |
| **Length of stay** (only for regular inpatient hospitalization) *(days)* | 2.00 [1.00; 4.00] | 2.00 [1.00; 3.00] | 2.00 [1.00; 3.00] | 2.00 [1.00; 3.00] | 2.00 [1.00; 3.00] | 2.00 [1.00; 3.00] | 2.00 [1.00; 3.00] | 2.00 [1.00; 4.00] | 2.00 [1.00; 3.00] | 2.00 [1.00; 3.00] | 2.00 [1.00; 3.00] | 2.00 [1.00; 3.25] | 2.00 [1.00; 4.00] |
| **Discharge modalities:** *Exitus* | 1% ( 1) | 0% ( 0) | 1% ( 1) | 0% ( 0) | 1% ( 1) | 1% ( 1) | 1% ( 1) | 0% ( 0) | 0% ( 0) | 1% ( 1) | 0% ( 0) | 1% ( 1) | 1% ( 1) |
| *Home* | 78% ( 58) | 84% ( 120) | 77% ( 100) | 84% ( 106) | 78% ( 97) | 78% ( 104) | 78% ( 122) | 86% ( 85) | 82% ( 77) | 80% ( 99) | 87% ( 61) | 87% ( 84) | 93% ( 79) |
| *Nursing home* | 0% ( 0) | 1% ( 1) | 1% ( 1) | 1% ( 1) | 0% ( 0) | 0% ( 0) | 0% ( 0) | 0% ( 0) | 0% ( 0) | 0% ( 0) | 0% ( 0) | 0% ( 0) | 0% ( 0) |
| *Home hospitalization* | 0% ( 0) | 0% ( 0) | 1% ( 1) | 2% ( 2) | 0% ( 0) | 1% ( 1) | 1% ( 1) | 0% ( 0) | 0% ( 0) | 2% ( 2) | 0% ( 0) | 0% ( 0) | 0% ( 0) |
| *Discharge against medical advice* | 16% ( 12) | 8% ( 11) | 8% ( 11) | 8% ( 10) | 14% ( 18) | 8% ( 11) | 10% ( 15) | 8% ( 8) | 13% ( 12) | 5% ( 6) | 7% ( 5) | 7% ( 7) | 2% ( 2) |
| *Transfer to another hospital* | 4% ( 3) | 8% ( 11) | 12% ( 16) | 5% ( 6) | 7% ( 9) | 12% ( 16) | 10% ( 16) | 6% ( 6) | 5% ( 5) | 11% ( 14) | 6% ( 4) | 5% ( 5) | 4% ( 3) |
| *Transfer in the same hospital* | 0% ( 0) | 0% ( 0) | 0% ( 0) | 1% ( 1) | 0% ( 0) | 1% ( 1) | 1% ( 1) | 0% ( 0) | 0% ( 0) | 1% ( 1) | 0% ( 0) | 0% ( 0) | 0% ( 0) |
| *Rehabilitation* | 0% ( 0) | 0% ( 0) | 0% ( 0) | 0% ( 0) | 0% ( 0) | 0% ( 0) | 0% ( 0) | 0% ( 0) | 0% ( 0) | 0% ( 0) | 0% ( 0) | 0% ( 0) | 0% ( 0) |
| *Home care assistance* | 0% ( 0) | 0% ( 0) | 0% ( 0) | 0% ( 0) | 0% ( 0) | 0% ( 0) | 0% ( 0) | 0% ( 0) | 0% ( 0) | 0% ( 0) | 0% ( 0) | 0% ( 0) | 0% ( 0) |
| **Hospitalization type:** *Newborn* | 0% ( 0) | 0% ( 0) | 0% ( 0) | 0% ( 0) | 0% ( 0) | 0% ( 0) | 0% ( 0) | 0% ( 0) | 0% ( 0) | 0% ( 0) | 0% ( 0) | 0% ( 0) | 0% ( 0) |
| *Non-urgent hospitalization* | 11% ( 8) | 11% ( 16) | 14% ( 18) | 7% ( 9) | 10% ( 13) | 5% ( 7) | 10% ( 16) | 11% ( 10) | 3% ( 3) | 8% ( 10) | 7% ( 5) | 9% ( 9) | 12% ( 10) |
| *Urgent hospitalization* | 89% ( 65) | 89% ( 125) | 82% ( 102) | 89% ( 108) | 90% ( 112) | 95% ( 125) | 88% ( 137) | 89% ( 85) | 97% ( 87) | 92% ( 112) | 93% ( 64) | 91% ( 86) | 88% ( 72) |
| *Planned hospitalization* | 0% ( 0) | 0% ( 0) | 0% ( 0) | 0% ( 0) | 0% ( 0) | 0% ( 0) | 0% ( 0) | 0% ( 0) | 0% ( 0) | 0% ( 0) | 0% ( 0) | 0% ( 0) | 0% ( 0) |
| *Other* | 0% ( 0) | 0% ( 0) | 4% ( 5) | 3% ( 4) | 0% ( 0) | 0% ( 0) | 1% ( 2) | 0% ( 0) | 0% ( 0) | 0% ( 0) | 0% ( 0) | 0% ( 0) | 0% ( 0) |
| **Trauma:** *Other* | 80% ( 12) | 90% ( 38) | 81% ( 44) | 80% ( 36) | 82% ( 40) | 61% ( 35) | 72% ( 41) | 78% ( 29) | 77% ( 27) | 73% ( 38) | 60% ( 18) | 56% ( 22) | 58% ( 15) |
| *Work-related injury* | 0% ( 0) | 0% ( 0) | 0% ( 0) | 0% ( 0) | 0% ( 0) | 2% ( 1) | 0% ( 0) | 0% ( 0) | 0% ( 0) | 0% ( 0) | 0% ( 0) | 0% ( 0) | 0% ( 0) |
| *Home accident* | 20% ( 3) | 10% ( 4) | 17% ( 9) | 20% ( 9) | 18% ( 9) | 33% ( 19) | 26% ( 15) | 22% ( 8) | 23% ( 8) | 27% ( 14) | 37% ( 11) | 44% ( 17) | 42% ( 11) |
| *Car accident* | 0% ( 0) | 0% ( 0) | 0% ( 0) | 0% ( 0) | 0% ( 0) | 0% ( 0) | 2% ( 1) | 0% ( 0) | 0% ( 0) | 0% ( 0) | 0% ( 0) | 0% ( 0) | 0% ( 0) |
| *Violence* | 0% ( 0) | 0% ( 0) | 2% ( 1) | 0% ( 0) | 0% ( 0) | 0% ( 0) | 0% ( 0) | 0% ( 0) | 0% ( 0) | 0% ( 0) | 0% ( 0) | 0% ( 0) | 0% ( 0) |
| *Self-inflicted injury* | 0% ( 0) | 0% ( 0) | 0% ( 0) | 0% ( 0) | 0% ( 0) | 4% ( 2) | 0% ( 0) | 0% ( 0) | 0% ( 0) | 0% ( 0) | 3% ( 1) | 0% ( 0) | 0% ( 0) |
| **DRG type:** medical | 99% ( 73) | 98% ( 140) | 100% ( 130) | 95% ( 120) | 99% ( 124) | 99% ( 133) | 99% ( 154) | 97% ( 96) | 98% ( 92) | 99% ( 122) | 96% ( 67) | 99% ( 96) | 98% ( 83) |
| **Nationality**: |  |  |  |  |  |  |  |  |  |  |  |  |  |
| *Italian* | 99% ( 73) | 97% ( 138) | 90% ( 117) | 93% ( 117) | 92% ( 115) | 93% ( 124) | 88% ( 137) | 84% ( 83) | 85% ( 80) | 88% ( 108) | 90% ( 63) | 88% ( 85) | 81% ( 69) |
| *Albanian* | 0% ( 0) | 1% ( 1) | 1% ( 1) | 2% ( 3) | 2% ( 2) | 1% ( 2) | 2% ( 3) | 2% ( 2) | 1% ( 1) | 2% ( 2) | 1% ( 1) | 1% ( 1) | 0% ( 0) |
| *Romanian* | 0% ( 0) | 1% ( 2) | 0% ( 0) | 2% ( 2) | 2% ( 2) | 1% ( 1) | 3% ( 5) | 6% ( 6) | 2% ( 2) | 5% ( 6) | 1% ( 1) | 6% ( 6) | 7% ( 6) |
| *Bengali* | 0% ( 0) | 0% ( 0) | 1% ( 1) | 0% ( 0) | 0% ( 0) | 0% ( 0) | 0% ( 0) | 0% ( 0) | 0% ( 0) | 0% ( 0) | 4% ( 3) | 1% ( 1) | 2% ( 2) |
| *Moroccan* | 0% ( 0) | 0% ( 0) | 2% ( 3) | 1% ( 1) | 1% ( 1) | 2% ( 3) | 2% ( 3) | 2% ( 2) | 5% ( 5) | 1% ( 1) | 0% ( 0) | 1% ( 1) | 1% ( 1) |
| **Main procedure:** |  |  |  |  |  |  |  |  |  |  |  |  |  |
| *Laryngoscopy and other tracheoscopy* | 6% ( 2) | 1% ( 1) | 2% ( 2) | 1% ( 1) | 1% ( 1) | 4% ( 3) | 5% ( 5) | 4% ( 3) | 4% ( 3) | 8% ( 7) | 4% ( 2) | 12% ( 9) | 6% ( 4) |
| *Fiber-optic bronchoscopy* | 9% ( 3) | 20% ( 16) | 10% ( 8) | 14% ( 13) | 10% ( 8) | 10% ( 8) | 15% ( 17) | 22% ( 16) | 23% ( 17) | 20% ( 18) | 19% ( 11) | 14% ( 11) | 21% ( 13) |
| *Other bronchoscopy* | 3% ( 1) | 11% ( 9) | 14% ( 11) | 8% ( 7) | 13% ( 11) | 14% ( 12) | 15% ( 16) | 8% ( 6) | 7% ( 5) | 14% ( 13) | 11% ( 6) | 6% ( 5) | 0% ( 0) |
| *Routine chest x-ray* | 16% ( 5) | 6% ( 5) | 15% ( 12) | 9% ( 8) | 12% ( 10) | 12% ( 10) | 13% ( 14) | 12% ( 9) | 11% ( 8) | 11% ( 10) | 11% ( 6) | 6% ( 5) | 3% ( 2) |
| *Other chest x-ray* | 3% ( 1) | 1% ( 1) | 1% ( 1) | 5% ( 5) | 4% ( 3) | 2% ( 2) | 1% ( 1) | 1% ( 1) | 0% ( 0) | 4% ( 4) | 4% ( 2) | 1% ( 1) | 2% ( 1) |
| *Microscopic examination of blood, other microscopic examination* | 0% ( 0) | 0% ( 0) | 0% ( 0) | 2% ( 2) | 1% ( 1) | 2% ( 2) | 5% ( 5) | 0% ( 0) | 4% ( 3) | 0% ( 0) | 2% ( 1) | 3% ( 2) | 3% ( 2) |
| *Removal of intraluminal FB rom pharynx without incision* | 6% ( 2) | 6% ( 5) | 7% ( 6) | 3% ( 3) | 7% ( 6) | 5% ( 4) | 5% ( 5) | 8% ( 6) | 3% ( 2) | 6% ( 5) | 7% ( 4) | 1% ( 1) | 10% ( 6) |
| *Removal of intraluminal FB from trachea and bronchus without incision* | 44% ( 14) | 27% ( 21) | 27% ( 22) | 32% ( 30) | 33% ( 27) | 31% ( 26) | 21% ( 23) | 24% ( 18) | 27% ( 20) | 16% ( 14) | 18% ( 10) | 23% ( 18) | 37% ( 23) |
| **Secondary procedure (1):** | 34% ( 12) | 28% ( 21) | 34% ( 21) | 49% ( 35) | 38% ( 23) | 33% ( 23) | 45% ( 39) | 51% ( 31) | 30% ( 17) | 38% ( 31) | 54% ( 25) | 39% ( 27) | 26% ( 18) |
| *Laryngoscopy and other tracheoscopy* | 3% ( 1) | 1% ( 1) | 2% ( 1) | 1% ( 1) | 2% ( 1) | 4% ( 3) | 1% ( 1) | 0% ( 0) | 5% ( 3) | 1% ( 1) | 0% ( 0) | 10% ( 7) | 13% ( 9) |
| *Fiber-optic bronchoscopy* | 20% ( 7) | 9% ( 7) | 3% ( 2) | 3% ( 2) | 3% ( 2) | 7% ( 5) | 5% ( 4) | 3% ( 2) | 11% ( 6) | 14% ( 11) | 11% ( 5) | 23% ( 16) | 20% ( 14) |
| *Other bronchoscopy* | 14% ( 5) | 7% ( 5) | 11% ( 7) | 6% ( 4) | 10% ( 6) | 10% ( 7) | 9% ( 8) | 3% ( 2) | 14% ( 8) | 2% ( 2) | 4% ( 2) | 7% ( 5) | 10% ( 7) |
| *Closed [endoscopic] biopsy of bronchus* | 0% ( 0) | 7% ( 5) | 2% ( 1) | 6% ( 4) | 12% ( 7) | 4% ( 3) | 2% ( 2) | 2% ( 1) | 0% ( 0) | 0% ( 0) | 0% ( 0) | 0% ( 0) | 1% ( 1) |
| *Routine chest x-ray* | 11% ( 4) | 15% ( 11) | 29% ( 18) | 14% ( 10) | 18% ( 11) | 19% ( 13) | 21% ( 18) | 25% ( 15) | 19% ( 11) | 16% ( 13) | 13% ( 6) | 14% ( 10) | 14% ( 10) |
| *Other chest x-ray* | 0% ( 0) | 9% ( 7) | 6% ( 4) | 4% ( 3) | 5% ( 3) | 6% ( 4) | 5% ( 4) | 5% ( 3) | 4% ( 2) | 2% ( 2) | 0% ( 0) | 3% ( 2) | 0% ( 0) |
| *Microscopic examination of blood, other microscopic examination* | 0% ( 0) | 8% ( 6) | 3% ( 2) | 6% ( 4) | 2% ( 1) | 0% ( 0) | 2% ( 2) | 3% ( 2) | 5% ( 3) | 2% ( 2) | 2% ( 1) | 4% ( 3) | 3% ( 2) |
| *Removal of intraluminal FB from trachea and bronchus without incision* | 17% ( 6) | 15% ( 11) | 10% ( 6) | 11% ( 8) | 10% ( 6) | 16% ( 11) | 10% ( 9) | 8% ( 5) | 12% ( 7) | 23% ( 19) | 15% ( 7) | 0% ( 0) | 12% ( 8) |

Table S7. Characteristics of hospitalization in female toddlers

|  | **2001** | **2002** | **2003** | **2004** | **2005** | **2006** | **2007** | **2008** | **2009** | **2010** | **2011** | **2012** | **2013** |
| --- | --- | --- | --- | --- | --- | --- | --- | --- | --- | --- | --- | --- | --- |
|  | **(N=49)** | **(N=92)** | **(N=76)** | **(N=76)** | **(N=83)** | **(N=70)** | **(N=91)** | **(N=62)** | **(N=81)** | **(N=85)** | **(N=52)** | **(N=61)** | **(N=46)** |
| **Age** *(years)*: 1 | 29% ( 14) | 28% ( 26) | 47% ( 36) | 55% ( 42) | 52% ( 43) | 67% ( 47) | 55% ( 50) | 53% ( 33) | 69% ( 56) | 58% ( 49) | 54% ( 28) | 52% ( 32) | 63% ( 29) |
| 2 | 53% ( 26) | 47% ( 43) | 36% ( 27) | 30% ( 23) | 36% ( 30) | 24% ( 17) | 26% ( 24) | 29% ( 18) | 20% ( 16) | 28% ( 24) | 29% ( 15) | 39% ( 24) | 20% ( 9) |
| 3 | 18% ( 9) | 25% ( 23) | 17% ( 13) | 14% ( 11) | 12% ( 10) | 9% ( 6) | 19% ( 17) | 18% ( 11) | 11% ( 9) | 14% ( 12) | 17% ( 9) | 8% ( 5) | 17% ( 8) |
| **Hospitalization:** *Day hospital service* | 2% ( 1) | 3% ( 3) | 4% ( 3) | 3% ( 2) | 1% ( 1) | 0% ( 0) | 2% ( 2) | 3% ( 2) | 5% ( 4) | 1% ( 1) | 2% ( 1) | 3% ( 2) | 2% ( 1) |
| **Length of stay** (only for regular inpatient hospitalization) *(days)* | 2.00 [1.00; 3.25] | 2.00 [1.00; 3.00] | 2.00 [1.00; 3.00] | 2.00 [1.00; 3.00] | 2.00 [1.00; 3.00] | 2.00 [1.00; 3.00] | 1.00 [1.00; 3.00] | 2.00 [1.00; 3.00] | 2.00 [1.00; 4.00] | 2.00 [1.00; 3.00] | 1.00 [1.00; 3.00] | 2.00 [1.00; 3.00] | 2.00 [1.00; 6.00] |
| **Discharge modalities:** *Exitus* | 0% ( 0) | 1% ( 1) | 0% ( 0) | 1% ( 1) | 0% ( 0) | 0% ( 0) | 1% ( 1) | 0% ( 0) | 0% ( 0) | 0% ( 0) | 2% ( 1) | 0% ( 0) | 0% ( 0) |
| *Home* | 71% ( 35) | 75% ( 69) | 80% ( 61) | 80% ( 61) | 81% ( 67) | 81% ( 57) | 81% ( 74) | 81% ( 50) | 83% ( 67) | 81% ( 69) | 81% ( 42) | 90% ( 55) | 78% ( 36) |
| *Nursing home* | 0% ( 0) | 0% ( 0) | 0% ( 0) | 0% ( 0) | 0% ( 0) | 0% ( 0) | 0% ( 0) | 0% ( 0) | 0% ( 0) | 0% ( 0) | 2% ( 1) | 0% ( 0) | 0% ( 0) |
| *Home hospitalization* | 2% ( 1) | 0% ( 0) | 1% ( 1) | 0% ( 0) | 4% ( 3) | 0% ( 0) | 0% ( 0) | 0% ( 0) | 0% ( 0) | 0% ( 0) | 0% ( 0) | 0% ( 0) | 2% ( 1) |
| *Discharge against medical advice* | 22% ( 11) | 13% ( 12) | 14% ( 11) | 14% ( 11) | 10% ( 8) | 7% ( 5) | 13% ( 12) | 6% ( 4) | 6% ( 5) | 7% ( 6) | 12% ( 6) | 3% ( 2) | 11% ( 5) |
| *Transfer to another hospital* | 4% ( 2) | 11% ( 10) | 3% ( 2) | 3% ( 2) | 6% ( 5) | 11% ( 8) | 4% ( 4) | 11% ( 7) | 11% ( 9) | 12% ( 10) | 4% ( 2) | 7% ( 4) | 9% ( 4) |
| *Transfer in the same hospital* | 0% ( 0) | 0% ( 0) | 1% ( 1) | 1% ( 1) | 0% ( 0) | 0% ( 0) | 0% ( 0) | 2% ( 1) | 0% ( 0) | 0% ( 0) | 0% ( 0) | 0% ( 0) | 0% ( 0) |
| *Rehabilitation* | 0% ( 0) | 0% ( 0) | 0% ( 0) | 0% ( 0) | 0% ( 0) | 0% ( 0) | 0% ( 0) | 0% ( 0) | 0% ( 0) | 0% ( 0) | 0% ( 0) | 0% ( 0) | 0% ( 0) |
| *Home care assistance* | 0% ( 0) | 0% ( 0) | 0% ( 0) | 0% ( 0) | 0% ( 0) | 0% ( 0) | 0% ( 0) | 0% ( 0) | 0% ( 0) | 0% ( 0) | 0% ( 0) | 0% ( 0) | 0% ( 0) |
| **Hospitalization type:** *Newborn* | 0% ( 0) | 0% ( 0) | 0% ( 0) | 0% ( 0) | 0% ( 0) | 0% ( 0) | 0% ( 0) | 0% ( 0) | 0% ( 0) | 0% ( 0) | 0% ( 0) | 0% ( 0) | 0% ( 0) |
| *Non-urgent hospitalization* | 21% ( 10) | 14% ( 13) | 14% ( 10) | 8% ( 6) | 8% ( 7) | 4% ( 3) | 4% ( 4) | 3% ( 2) | 8% ( 6) | 4% ( 3) | 10% ( 5) | 7% ( 4) | 4% ( 2) |
| *Urgent hospitalization* | 79% ( 38) | 86% ( 79) | 84% ( 61) | 89% ( 66) | 92% ( 76) | 96% ( 67) | 96% ( 85) | 95% ( 58) | 92% ( 71) | 96% ( 80) | 90% ( 46) | 93% ( 54) | 96% ( 43) |
| *Planned hospitalization* | 0% ( 0) | 0% ( 0) | 0% ( 0) | 0% ( 0) | 0% ( 0) | 0% ( 0) | 0% ( 0) | 0% ( 0) | 0% ( 0) | 0% ( 0) | 0% ( 0) | 0% ( 0) | 0% ( 0) |
| *Other* | 0% ( 0) | 0% ( 0) | 3% ( 2) | 3% ( 2) | 0% ( 0) | 0% ( 0) | 0% ( 0) | 2% ( 1) | 0% ( 0) | 0% ( 0) | 0% ( 0) | 0% ( 0) | 0% ( 0) |
| **Trauma:** *Other* | 100% ( 15) | 79% ( 23) | 81% ( 22) | 76% ( 16) | 78% ( 31) | 57% ( 13) | 59% ( 16) | 100% ( 21) | 77% ( 30) | 79% ( 30) | 75% ( 18) | 48% ( 12) | 65% ( 11) |
| *Work-related injury* | 0% ( 0) | 0% ( 0) | 0% ( 0) | 5% ( 1) | 0% ( 0) | 0% ( 0) | 0% ( 0) | 0% ( 0) | 0% ( 0) | 0% ( 0) | 0% ( 0) | 0% ( 0) | 0% ( 0) |
| *Home accident* | 0% ( 0) | 21% ( 6) | 19% ( 5) | 19% ( 4) | 22% ( 9) | 43% ( 10) | 41% ( 11) | 0% ( 0) | 23% ( 9) | 21% ( 8) | 25% ( 6) | 48% ( 12) | 35% ( 6) |
| *Car accident* | 0% ( 0) | 0% ( 0) | 0% ( 0) | 0% ( 0) | 0% ( 0) | 0% ( 0) | 0% ( 0) | 0% ( 0) | 0% ( 0) | 0% ( 0) | 0% ( 0) | 0% ( 0) | 0% ( 0) |
| *Violence* | 0% ( 0) | 0% ( 0) | 0% ( 0) | 0% ( 0) | 0% ( 0) | 0% ( 0) | 0% ( 0) | 0% ( 0) | 0% ( 0) | 0% ( 0) | 0% ( 0) | 0% ( 0) | 0% ( 0) |
| *Self-inflicted injury* | 0% ( 0) | 0% ( 0) | 0% ( 0) | 0% ( 0) | 0% ( 0) | 0% ( 0) | 0% ( 0) | 0% ( 0) | 0% ( 0) | 0% ( 0) | 0% ( 0) | 4% ( 1) | 0% ( 0) |
| **DRG type:** medical | 100% ( 49) | 98% ( 90) | 99% ( 75) | 100% ( 76) | 96% ( 80) | 100% ( 70) | 99% ( 90) | 95% ( 59) | 100% ( 81) | 100% ( 85) | 94% ( 49) | 100% ( 61) | 98% ( 45) |
| **Nationality**: *Italian* | 100% ( 49) | 97% ( 89) | 91% ( 68) | 91% ( 69) | 94% ( 78) | 84% ( 59) | 88% ( 80) | 85% ( 53) | 88% ( 71) | 85% ( 72) | 79% ( 41) | 87% ( 53) | 78% ( 36) |
| *Albanian* | 0% ( 0) | 0% ( 0) | 0% ( 0) | 3% ( 2) | 1% ( 1) | 1% ( 1) | 3% ( 3) | 0% ( 0) | 2% ( 2) | 2% ( 2) | 2% ( 1) | 2% ( 1) | 0% ( 0) |
| *Romanian* | 0% ( 0) | 0% ( 0) | 0% ( 0) | 0% ( 0) | 0% ( 0) | 3% ( 2) | 4% ( 4) | 2% ( 1) | 2% ( 2) | 5% ( 4) | 6% ( 3) | 2% ( 1) | 11% ( 5) |
| *Indian* | 0% ( 0) | 0% ( 0) | 0% ( 0) | 1% ( 1) | 0% ( 0) | 0% ( 0) | 0% ( 0) | 2% ( 1) | 4% ( 3) | 1% ( 1) | 2% ( 1) | 0% ( 0) | 0% ( 0) |
| *Moroccan* | 0% ( 0) | 1% ( 1) | 4% ( 3) | 3% ( 2) | 4% ( 3) | 9% ( 6) | 1% ( 1) | 5% ( 3) | 0% ( 0) | 2% ( 2) | 10% ( 5) | 7% ( 4) | 2% ( 1) |
| **Main procedure :** |  |  |  |  |  |  |  |  |  |  |  |  |  |
| *Laryngoscopy and other tracheoscopy* | 0% ( 0) | 2% ( 1) | 4% ( 2) | 3% ( 2) | 2% ( 1) | 0% ( 0) | 2% ( 1) | 9% ( 5) | 2% ( 1) | 9% ( 5) | 10% ( 4) | 16% ( 7) | 12% ( 4) |
| *Fiber-optic bronchoscopy* | 0% ( 0) | 25% ( 12) | 23% ( 12) | 10% ( 6) | 11% ( 6) | 16% ( 7) | 6% ( 4) | 15% ( 8) | 25% ( 15) | 15% ( 8) | 15% ( 6) | 23% ( 10) | 18% ( 6) |
| *Other bronchoscopy* | 11% ( 2) | 4% ( 2) | 10% ( 5) | 10% ( 6) | 12% ( 7) | 23% ( 10) | 16% ( 10) | 9% ( 5) | 15% ( 9) | 13% ( 7) | 10% ( 4) | 2% ( 1) | 9% ( 3) |
| *Closed [endoscopic] biopsy of bronchus* | 5% ( 1) | 0% ( 0) | 4% ( 2) | 3% ( 2) | 2% ( 1) | 2% ( 1) | 2% ( 1) | 4% ( 2) | 2% ( 1) | 6% ( 3) | 0% ( 0) | 2% ( 1) | 0% ( 0) |
| *Routine chest x-ray* | 0% ( 0) | 2% ( 1) | 12% ( 6) | 10% ( 6) | 9% ( 5) | 9% ( 4) | 11% ( 7) | 11% ( 6) | 8% ( 5) | 11% ( 6) | 5% ( 2) | 12% ( 5) | 3% ( 1) |
| *Other chest x-ray* | 5% ( 1) | 8% ( 4) | 6% ( 3) | 0% ( 0) | 0% ( 0) | 5% ( 2) | 0% ( 0) | 0% ( 0) | 0% ( 0) | 4% ( 2) | 0% ( 0) | 5% ( 2) | 0% ( 0) |
| *Removal of intraluminal FB from pharynx without incision* | 5% ( 1) | 10% ( 5) | 6% ( 3) | 5% ( 3) | 9% ( 5) | 9% ( 4) | 13% ( 8) | 5% ( 3) | 8% ( 5) | 6% ( 3) | 8% ( 3) | 9% ( 4) | 6% ( 2) |
| *Removal of intraluminal FB from trachea and bronchus without incision* | 47% ( 9) | 23% ( 11) | 23% ( 12) | 15% ( 9) | 28% ( 16) | 25% ( 11) | 21% ( 13) | 22% ( 12) | 15% ( 9) | 8% ( 4) | 21% ( 8) | 14% ( 6) | 6% ( 2) |
| **Secondary procedure (1) :** | 29% ( 8) | 34% ( 13) | 42% ( 13) | 44% ( 16) | 38% ( 15) | 41% ( 15) | 47% ( 24) | 30% ( 10) | 38% ( 19) | 53% ( 26) | 39% ( 13) | 36% ( 15) | 57% ( 20) |
| *Laryngoscopy and other tracheoscopy* | 7% ( 2) | 0% ( 0) | 3% ( 1) | 0% ( 0) | 3% ( 1) | 5% ( 2) | 4% ( 2) | 3% ( 1) | 8% ( 4) | 2% ( 1) | 3% ( 1) | 14% ( 6) | 14% ( 5) |
| *Fiber-optic bronchoscopy* | 4% ( 1) | 5% ( 2) | 0% ( 0) | 6% ( 2) | 10% ( 4) | 8% ( 3) | 6% ( 3) | 9% ( 3) | 2% ( 1) | 8% ( 4) | 6% ( 2) | 14% ( 6) | 6% ( 2) |
| *Other bronchoscopy* | 18% ( 5) | 8% ( 3) | 6% ( 2) | 11% ( 4) | 10% ( 4) | 0% ( 0) | 14% ( 7) | 9% ( 3) | 10% ( 5) | 4% ( 2) | 9% ( 3) | 5% ( 2) | 3% ( 1) |
| *Routine chest x-ray, so described* | 43% ( 12) | 29% ( 11) | 26% ( 8) | 25% ( 9) | 21% ( 8) | 16% ( 6) | 14% ( 7) | 15% ( 5) | 24% ( 12) | 18% ( 9) | 9% ( 3) | 10% ( 4) | 6% ( 2) |
| *Other chest x-ray* | 0% ( 0) | 13% ( 5) | 6% ( 2) | 0% ( 0) | 5% ( 2) | 11% ( 4) | 4% ( 2) | 6% ( 2) | 0% ( 0) | 0% ( 0) | 6% ( 2) | 2% ( 1) | 0% ( 0) |
| *Consultation, described as limited* | 0% ( 0) | 3% ( 1) | 3% ( 1) | 6% ( 2) | 8% ( 3) | 3% ( 1) | 2% ( 1) | 0% ( 0) | 6% ( 3) | 0% ( 0) | 0% ( 0) | 0% ( 0) | 0% ( 0) |
| *Microscopic examination of blood, other microscopic examination* | 0% ( 0) | 5% ( 2) | 0% ( 0) | 0% ( 0) | 3% ( 1) | 0% ( 0) | 2% ( 1) | 9% ( 3) | 0% ( 0) | 6% ( 3) | 6% ( 2) | 7% ( 3) | 3% ( 1) |
| *Removal of intraluminal FB from trachea and bronchus without incision* | 0% ( 0) | 3% ( 1) | 13% ( 4) | 8% ( 3) | 3% ( 1) | 16% ( 6) | 8% ( 4) | 18% ( 6) | 12% ( 6) | 8% ( 4) | 21% ( 7) | 12% ( 5) | 11% ( 4) |

Table S8. Characteristics of hospitalization in male children (4-14 years)

|  | **2001** | **2002** | **2003** | **2004** | **2005** | **2006** | **2007** | **2008** | **2009** | **2010** | **2011** | **2012** | **2013** |
| --- | --- | --- | --- | --- | --- | --- | --- | --- | --- | --- | --- | --- | --- |
|  | **(N=35)** | **(N=78)** | **(N=52)** | **(N=56)** | **(N=54)** | **(N=39)** | **(N=70)** | **(N=35)** | **(N=42)** | **(N=39)** | **(N=56)** | **(N=45)** | **(N=33)** |
| **Age** *(days)* | 8.00 [6.00; 11.00] | 6.00/ [5.00; 9.75] | 6.00 [4.75; 10.25] | 7.00 [5.00; 10.00] | 6.50 [5.00; 11.00] | 7.00 [5.00; 10.00] | 7.00 [5.00; 10.00] | 7.00 [5.00; 11.00] | 6.00 [5.00; 10.00] | 8.00 [5.00; 11.00] | 7.00 [4.0; 10.25] | 7.00 [5.00: 10.00] | 7.00 [5.00: 10.00] 7.00 [5.00: 10.00] |
| **Hospitalization:** *Day hospital service* | 0% ( 0) | 3% ( 2) | 6% ( 3) | 7% ( 4) | 2% ( 1) | 3% ( 1) | 7% ( 5) | 9% ( 3) | 7% ( 3) | 15% ( 6) | 7% ( 4) | 7% ( 3) | 9% ( 3) |
| **Length of stay** (only for regular inpatient hospitalization) *(days)* | 2.00 [1.00; 2.00] | 2.00 [1.00; 3.00] | 0.00/1.00/2.00 | 1.00 [1.00; 3.00] | 1.00 [1.00; 2.00] | 1.00 [1.00; 2.00] | 1.00 [1.00; 2.00] | 1.00 [1.00; 2.25] | 1.00 [1.00; 2.50] | 1.00 [1.00; 2.00] | 1.00 [1.00; 3.00] | 1.00 [1.00; 2.00] | 1.00 [1.00; 2.00] |
| **Discharge modalities:** *Exitus* | 0% ( 0) | 0% ( 0) | 0% ( 0) | 0% ( 0) | 0% ( 0) | 0% ( 0) | 1% ( 1) | 0% ( 0) | 0% ( 0) | 0% ( 0) | 0% ( 0) | 2% ( 1) | 0% ( 0) |
| *Home* | 63% ( 22) | 85% ( 66) | 73% ( 38) | 80% ( 45) | 80% ( 43) | 87% ( 34) | 87% ( 61) | 94% ( 33) | 90% ( 38) | 90% ( 35) | 79% ( 44) | 89% ( 40) | 82% ( 27) |
| *Nursing home* | 0% ( 0) | 0% ( 0) | 0% ( 0) | 0% ( 0) | 0% ( 0) | 0% ( 0) | 0% ( 0) | 0% ( 0) | 2% ( 1) | 0% ( 0) | 0% ( 0) | 0% ( 0) | 0% ( 0) |
| *Home hospitalization* | 3% ( 1) | 0% ( 0) | 2% ( 1) | 0% ( 0) | 0% ( 0) | 0% ( 0) | 0% ( 0) | 0% ( 0) | 0% ( 0) | 0% ( 0) | 0% ( 0) | 0% ( 0) | 3% ( 1) |
| *Discharge against medical advice* | 34% ( 12) | 10% ( 8) | 17% ( 9) | 18% ( 10) | 13% ( 7) | 8% ( 3) | 9% ( 6) | 6% ( 2) | 5% ( 2) | 5% ( 2) | 14% ( 8) | 7% ( 3) | 6% ( 2) |
| *Transfer to another hospital* | 0% ( 0) | 5% ( 4) | 8% ( 4) | 2% ( 1) | 7% ( 4) | 5% ( 2) | 3% ( 2) | 0% ( 0) | 2% ( 1) | 5% ( 2) | 7% ( 4) | 2% ( 1) | 6% ( 2) |
| *Transfer in the same hospital* | 0% ( 0) | 0% ( 0) | 0% ( 0) | 0% ( 0) | 0% ( 0) | 0% ( 0) | 0% ( 0) | 0% ( 0) | 0% ( 0) | 0% ( 0) | 0% ( 0) | 0% ( 0) | 3% ( 1) |
| *Rehabilitation* | 0% ( 0) | 0% ( 0) | 0% ( 0) | 0% ( 0) | 0% ( 0) | 0% ( 0) | 0% ( 0) | 0% ( 0) | 0% ( 0) | 0% ( 0) | 0% ( 0) | 0% ( 0) | 0% ( 0) |
| *Home care assistance* | 0% ( 0) | 0% ( 0) | 0% ( 0) | 0% ( 0) | 0% ( 0) | 0% ( 0) | 0% ( 0) | 0% ( 0) | 0% ( 0) | 0% ( 0) | 0% ( 0) | 0% ( 0) | 0% ( 0) |
| **Hospitalization type:** *Newborn* | 0% ( 0) | 0% ( 0) | 0% ( 0) | 0% ( 0) | 0% ( 0) | 0% ( 0) | 0% ( 0) | 3% ( 1) | 0% ( 0) | 0% ( 0) | 0% ( 0) | 0% ( 0) | 0% ( 0) |
| *Non-urgent hospitalization* | 6% ( 2) | 10% ( 8) | 16% ( 8) | 13% ( 7) | 11% ( 6) | 5% ( 2) | 5% ( 3) | 0% ( 0) | 8% ( 3) | 9% ( 3) | 9% ( 5) | 0% ( 0) | 16% ( 5) |
| *Urgent hospitalization* | 94% ( 33) | 90% ( 69) | 82% ( 41) | 85% ( 44) | 89% ( 47) | 95% ( 36) | 94% ( 61) | 97% ( 32) | 92% ( 36) | 91% ( 30) | 91% ( 48) | 100% ( 42) | 84% ( 26) |
| *Planned hospitalization* | 0% ( 0) | 0% ( 0) | 0% ( 0) | 0% ( 0) | 0% ( 0) | 0% ( 0) | 2% ( 1) | 0% ( 0) | 0% ( 0) | 0% ( 0) | 0% ( 0) | 0% ( 0) | 0% ( 0) |
| *Other* | 0% ( 0) | 0% ( 0) | 2% ( 1) | 2% ( 1) | 0% ( 0) | 0% ( 0) | 0% ( 0) | 0% ( 0) | 0% ( 0) | 0% ( 0) | 0% ( 0) | 0% ( 0) | 0% ( 0) |
| **Trauma:** *Other* | 100% ( 12) | 92% ( 23) | 77% ( 17) | 76% ( 13) | 73% ( 16) | 83% ( 15) | 65% ( 17) | 86% ( 12) | 100% ( 17) | 90% ( 18) | 50% ( 9) | 89% ( 17) | 65% ( 11) |
| *Work-related injury* | 0% ( 0) | 0% ( 0) | 0% ( 0) | 6% ( 1) | 0% ( 0) | 0% ( 0) | 0% ( 0) | 0% ( 0) | 0% ( 0) | 0% ( 0) | 0% ( 0) | 0% ( 0) | 0% ( 0) |
| *Home accident* | 0% ( 0) | 8% ( 2) | 23% ( 5) | 18% ( 3) | 27% ( 6) | 11% ( 2) | 35% ( 9) | 14% ( 2) | 0% ( 0) | 10% ( 2) | 50% ( 9) | 11% ( 2) | 35% ( 6) |
| *Car accident* | 0% ( 0) | 0% ( 0) | 0% ( 0) | 0% ( 0) | 0% ( 0) | 0% ( 0) | 0% ( 0) | 0% ( 0) | 0% ( 0) | 0% ( 0) | 0% ( 0) | 0% ( 0) | 0% ( 0) |
| *Violence* | 0% ( 0) | 0% ( 0) | 0% ( 0) | 0% ( 0) | 0% ( 0) | 0% ( 0) | 0% ( 0) | 0% ( 0) | 0% ( 0) | 0% ( 0) | 0% ( 0) | 0% ( 0) | 0% ( 0) |
| *Self-inflicted injury* | 0% ( 0) | 0% ( 0) | 0% ( 0) | 0% ( 0) | 0% ( 0) | 6% ( 1) | 0% ( 0) | 0% ( 0) | 0% ( 0) | 0% ( 0) | 0% ( 0) | 0% ( 0) | 0% ( 0) |
| **DRG type:** medical | 100% ( 35) | 94% ( 73) | 98% ( 51) | 96% ( 54) | 98% ( 53) | 97% ( 38) | 99% ( 69) | 91% ( 32) | 98% ( 41) | 95% ( 37) | 98% ( 55) | 98% ( 44) | 100% ( 33) |
| **Nationality:** *Italian* | 100% ( 35) | 99% ( 77) | 100% ( 52) | 95% ( 53) | 91% ( 49) | 95% ( 37) | 93% ( 65) | 91% ( 32) | 90% ( 37) | 90% ( 35) | 88% ( 49) | 93% ( 42) | 82% ( 27) |
| *Albanian* | 0% ( 0) | 0% ( 0) | 0% ( 0) | 4% ( 2) | 2% ( 1) | 0% ( 0) | 0% ( 0) | 0% ( 0) | 2% ( 1) | 3% ( 1) | 4% ( 2) | 2% ( 1) | 0% ( 0) |
| *Romanian* | 0% ( 0) | 0% ( 0) | 0% ( 0) | 0% ( 0) | 0% ( 0) | 0% ( 0) | 0% ( 0) | 3% ( 1) | 2% ( 1) | 0% ( 0) | 4% ( 2) | 0% ( 0) | 0% ( 0) |
| *Moroccan* | 0% ( 0) | 0% ( 0) | 0% ( 0) | 0% ( 0) | 0% ( 0) | 0% ( 0) | 1% ( 1) | 0% ( 0) | 2% ( 1) | 0% ( 0) | 2% ( 1) | 0% ( 0) | 3% ( 1) |
| **Main procedure:** |  |  |  |  |  |  |  |  |  |  |  |  |  |
| *Laryngoscopy and other tracheoscopy* | 16% ( 3) | 2% ( 1) | 9% ( 3) | 2% ( 1) | 3% ( 1) | 0% ( 0) | 13% ( 6) | 7% ( 2) | 14% ( 5) | 6% ( 2) | 11% ( 5) | 5% ( 2) | 4% ( 1) |
| *Fiber-optic bronchoscopy* | 0% ( 0) | 11% ( 5) | 24% ( 8) | 24% ( 10) | 10% ( 3) | 16% ( 4) | 13% ( 6) | 4% ( 1) | 6% ( 2) | 6% ( 2) | 9% ( 4) | 11% ( 4) | 16% ( 4) |
| *Other bronchoscopy* | 0% ( 0) | 9% ( 4) | 0% ( 0) | 15% ( 6) | 6% ( 2) | 4% ( 1) | 13% ( 6) | 4% ( 1) | 6% ( 2) | 13% ( 4) | 14% ( 6) | 8% ( 3) | 4% ( 1) |
| *Routine chest x-ray* | 11% ( 2) | 15% ( 7) | 21% ( 7) | 10% ( 4) | 10% ( 3) | 4% ( 1) | 4% ( 2) | 11% ( 3) | 3% ( 1) | 0% ( 0) | 5% ( 2) | 5% ( 2) | 12% ( 3) |
| *Microscopic examination of blood, other microscopic examination* | 0% ( 0) | 2% ( 1) | 0% ( 0) | 0% ( 0) | 6% ( 2) | 0% ( 0) | 0% ( 0) | 4% ( 1) | 0% ( 0) | 0% ( 0) | 5% ( 2) | 3% ( 1) | 4% ( 1) |
| *Removal of intraluminal FB from pharynx without incision* | 16% ( 3) | 17% ( 8) | 9% ( 3) | 7% ( 3) | 19% ( 6) | 36% ( 9) | 20% ( 9) | 0% ( 0) | 17% ( 6) | 13% ( 4) | 14% ( 6) | 8% ( 3) | 20% ( 5) |
| *Removal of intraluminal FB from larynx without incision* | 0% ( 0) | 0% ( 0) | 0% ( 0) | 2% ( 1) | 6% ( 2) | 0% ( 0) | 7% ( 3) | 0% ( 0) | 6% ( 2) | 6% ( 2) | 5% ( 2) | 0% ( 0) | 0% ( 0) |
| *Removal of intraluminal FB from trachea and bronchus without incision* | 32% ( 6) | 13% ( 6) | 3% ( 1) | 2% ( 1) | 16% ( 5) | 8% ( 2) | 11% ( 5) | 25% ( 7) | 14% ( 5) | 10% ( 3) | 11% ( 5) | 13% ( 5) | 16% ( 4) |
| **Secondary procedure (1):** |  |  |  |  |  |  |  |  |  |  |  |  |  |
| *Laryngoscopy and other tracheoscopy* | 0% ( 0) | 0% ( 0) | 4% ( 1) | 0% ( 0) | 0% ( 0) | 6% ( 1) | 11% ( 4) | 20% ( 5) | 9% ( 2) | 6% ( 1) | 10% ( 3) | 0% ( 0) | 11% ( 2) |
| *Fiber-optic bronchoscopy* | 8% ( 1) | 9% ( 3) | 7% ( 2) | 0% ( 0) | 8% ( 2) | 6% ( 1) | 3% ( 1) | 4% ( 1) | 9% ( 2) | 6% ( 1) | 7% ( 2) | 7% ( 2) | 11% ( 2) |
| *Other bronchoscopy* | 0% ( 0) | 6% ( 2) | 7% ( 2) | 0% ( 0) | 8% ( 2) | 6% ( 1) | 3% ( 1) | 4% ( 1) | 17% ( 4) | 12% ( 2) | 0% ( 0) | 10% ( 3) | 5% ( 1) |
| *Routine chest x-ray* | 33% ( 4) | 18% ( 6) | 11% ( 3) | 13% ( 3) | 28% ( 7) | 29% ( 5) | 11% ( 4) | 12% ( 3) | 13% ( 3) | 6% ( 1) | 3% ( 1) | 10% ( 3) | 16% ( 3) |
| *Electrocardiogram* | 0% ( 0) | 6% ( 2) | 4% ( 1) | 9% ( 2) | 4% ( 1) | 0% ( 0) | 5% ( 2) | 8% ( 2) | 0% ( 0) | 0% ( 0) | 3% ( 1) | 10% ( 3) | 5% ( 1) |
| *Microscopic examination of blood, other microscopic examination* | 8% ( 1) | 3% ( 1) | 7% ( 2) | 0% ( 0) | 0% ( 0) | 0% ( 0) | 5% ( 2) | 4% ( 1) | 4% ( 1) | 6% ( 1) | 7% ( 2) | 3% ( 1) | 5% ( 1) |
| *Removal of intraluminal FB from pharynx without incision* | 8% ( 1) | 0% ( 0) | 7% ( 2) | 0% ( 0) | 4% ( 1) | 6% ( 1) | 3% ( 1) | 0% ( 0) | 0% ( 0) | 6% ( 1) | 3% ( 1) | 3% ( 1) | 0% ( 0) |
| *Removal of intraluminal FB from trachea and bronchus without incision* | 8% ( 1) | 3% ( 1) | 7% ( 2) | 4% ( 1) | 4% ( 1) | 12% ( 2) | 5% ( 2) | 8% ( 2) | 0% ( 0) | 12% ( 2) | 20% ( 6) | 7% ( 2) | 11% ( 2) |

Table S9. Characteristics of hospitalization in female children (4-14 years)

|  | **2001** | **2002** | **2003** | **2004** | **2005** | **2006** | **2007** | **2008** | **2009** | **2010** | **2011** | **2012** | **2013** |
| --- | --- | --- | --- | --- | --- | --- | --- | --- | --- | --- | --- | --- | --- |
|  | **(N=15)** | **(N=46)** | **(N=39)** | **(N=39)** | **(N=25)** | **(N=39)** | **(N=42)** | **(N=29)** | **(N=28)** | **(N=18)** | **(N=22)** | **(N=18)** | **(N=25)** |
| **Age** *(days)* | 6.00 [5.00; 9.00] | 6.00 [4.25; 8.75] | 6.00 [4.50; 10.50] | 5.00 [4.00; 9.00] | 6.00 [4.00; 8.00] | 6.00 [4.00; 9.50] | 8.00 [5.00; 10.00] | 9.00 [5.00; 10.00] | 6.50 [4.00; 8.00] | 7.00 [5.25; 9.75] | 6.50 [5.00; 8.75] | 5.50 [5.50; 7.75] | 7.00 [5.00; 8.00] |
| **Hospitalization:** *Day hospital service* | 0% ( 0) | 4% ( 2) | 8% ( 3) | 8% ( 3) | 16% ( 4) | 8% ( 3) | 14% ( 6) | 0% ( 0) | 7% ( 2) | 0% ( 0) | 0% ( 0) | 11% ( 2) | 4% ( 1) |
| **Length of stay** (only for regular inpatient hospitalization) *(days)* | 1 [0; 2] | 1 [1; 2] | 1 [0; 2] | 1 [1; 2] | 1 [0; 2] | 1 [0; 2] | 1 [0; 2] | 2 [1; 3] | 1 [1; 2] | 2 [1; 2] | 1 [1; 2] | 2 [1; 2] | 1 [1; 2] |
| **Discharge modalities:** *Exitus* | 0% ( 0) | 0% ( 0) | 3% ( 1) | 0% ( 0) | 0% ( 0) | 3% ( 1) | 0% ( 0) | 3% ( 1) | 0% ( 0) | 0% ( 0) | 0% ( 0) | 0% ( 0) | 0% ( 0) |
| *Home* | 67% ( 10) | 89% ( 41) | 77% ( 30) | 85% ( 33) | 80% ( 20) | 85% ( 33) | 90% ( 38) | 83% ( 24) | 86% ( 24) | 100% ( 18) | 91% ( 20) | 94% ( 17) | 92% ( 23) |
| *Nursing home* | 0% ( 0) | 0% ( 0) | 0% ( 0) | 0% ( 0) | 0% ( 0) | 0% ( 0) | 0% ( 0) | 0% ( 0) | 0% ( 0) | 0% ( 0) | 0% ( 0) | 0% ( 0) | 0% ( 0) |
| *Home hospitalization* | 0% ( 0) | 2% ( 1) | 0% ( 0) | 0% ( 0) | 0% ( 0) | 0% ( 0) | 0% ( 0) | 0% ( 0) | 4% ( 1) | 0% ( 0) | 0% ( 0) | 0% ( 0) | 0% ( 0) |
| *Discharge against medical advice* | 27% ( 4) | 4% ( 2) | 13% ( 5) | 8% ( 3) | 20% ( 5) | 13% ( 5) | 5% ( 2) | 10% ( 3) | 0% ( 0) | 0% ( 0) | 5% ( 1) | 6% ( 1) | 8% ( 2) |
| *Transfer to another hospital* | 7% ( 1) | 4% ( 2) | 8% ( 3) | 8% ( 3) | 0% ( 0) | 0% ( 0) | 5% ( 2) | 0% ( 0) | 11% ( 3) | 0% ( 0) | 5% ( 1) | 0% ( 0) | 0% ( 0) |
| *Transfer in the same hospital* | 0% ( 0) | 0% ( 0) | 0% ( 0) | 0% ( 0) | 0% ( 0) | 0% ( 0) | 0% ( 0) | 0% ( 0) | 0% ( 0) | 0% ( 0) | 0% ( 0) | 0% ( 0) | 0% ( 0) |
| *Rehabilitation* | 0% ( 0) | 0% ( 0) | 0% ( 0) | 0% ( 0) | 0% ( 0) | 0% ( 0) | 0% ( 0) | 3% ( 1) | 0% ( 0) | 0% ( 0) | 0% ( 0) | 0% ( 0) | 0% ( 0) |
| *Home care assistance* | 0% ( 0) | 0% ( 0) | 0% ( 0) | 0% ( 0) | 0% ( 0) | 0% ( 0) | 0% ( 0) | 0% ( 0) | 0% ( 0) | 0% ( 0) | 0% ( 0) | 0% ( 0) | 0% ( 0) |
| **Hospitalization type:** *Newborn* | 0% ( 0) | 0% ( 0) | 0% ( 0) | 0% ( 0) | 0% ( 0) | 0% ( 0) | 0% ( 0) | 0% ( 0) | 0% ( 0) | 0% ( 0) | 0% ( 0) | 0% ( 0) | 0% ( 0) |
| *Non-urgent hospitalization* | 20% ( 3) | 11% ( 5) | 21% ( 8) | 14% ( 5) | 17% ( 4) | 14% ( 5) | 14% ( 5) | 14% ( 4) | 4% ( 1) | 22% ( 4) | 9% ( 2) | 25% ( 4) | 4% ( 1) |
| *Urgent hospitalization* | 80% ( 12) | 89% ( 39) | 79% ( 30) | 84% ( 31) | 83% ( 20) | 86% ( 32) | 86% ( 32) | 86% ( 25) | 96% ( 25) | 78% ( 14) | 91% ( 20) | 75% ( 12) | 96% ( 23) |
| *Planned hospitalization* | 0% ( 0) | 0% ( 0) | 0% ( 0) | 0% ( 0) | 0% ( 0) | 0% ( 0) | 0% ( 0) | 0% ( 0) | 0% ( 0) | 0% ( 0) | 0% ( 0) | 0% ( 0) | 0% ( 0) |
| *Other* | 0% ( 0) | 0% ( 0) | 0% ( 0) | 3% ( 1) | 0% ( 0) | 0% ( 0) | 0% ( 0) | 0% ( 0) | 0% ( 0) | 0% ( 0) | 0% ( 0) | 0% ( 0) | 0% ( 0) |
| **Trauma:** *Other* | 100% ( 4) | 81% ( 13) | 83% ( 10) | 80% ( 8) | 89% ( 8) | 75% ( 15) | 69% ( 9) | 64% ( 9) | 50% ( 6) | 73% ( 8) | 100% ( 10) | 40% ( 2) | 46% ( 6) |
| *Work-related injury* | 0% ( 0) | 0% ( 0) | 0% ( 0) | 0% ( 0) | 0% ( 0) | 0% ( 0) | 0% ( 0) | 0% ( 0) | 0% ( 0) | 0% ( 0) | 0% ( 0) | 0% ( 0) | 0% ( 0) |
| *Home accident* | 0% ( 0) | 19% ( 3) | 17% ( 2) | 20% ( 2) | 11% ( 1) | 25% ( 5) | 31% ( 4) | 36% ( 5) | 50% ( 6) | 27% ( 3) | 0% ( 0) | 60% ( 3) | 54% ( 7) |
| *Car accident* | 0% ( 0) | 0% ( 0) | 0% ( 0) | 0% ( 0) | 0% ( 0) | 0% ( 0) | 0% ( 0) | 0% ( 0) | 0% ( 0) | 0% ( 0) | 0% ( 0) | 0% ( 0) | 0% ( 0) |
| *Violence* | 0% ( 0) | 0% ( 0) | 0% ( 0) | 0% ( 0) | 0% ( 0) | 0% ( 0) | 0% ( 0) | 0% ( 0) | 0% ( 0) | 0% ( 0) | 0% ( 0) | 0% ( 0) | 0% ( 0) |
| *Self-inflicted injury* | 0% ( 0) | 0% ( 0) | 0% ( 0) | 0% ( 0) | 0% ( 0) | 0% ( 0) | 0% ( 0) | 0% ( 0) | 0% ( 0) | 0% ( 0) | 0% ( 0) | 0% ( 0) | 0% ( 0) |
| **DRG type:** medical | 100% ( 15) | 96% ( 44) | 95% ( 37) | 97% ( 38) | 88% ( 22) | 95% ( 37) | 95% ( 40) | 97% ( 28) | 96% ( 27) | 100% ( 18) | 95% ( 21) | 94% ( 17) | 96% ( 24) |
| **Nationality:** | 0% ( 0) | 0% ( 0) | 0% ( 0) | 0% ( 0) | 0% ( 0) | 0% ( 0) | 0% ( 0) | 0% ( 0) | 0% ( 0) | 0% ( 0) | 0% ( 0) | 0% ( 0) | 8% ( 2) |
| *Italian* | 100% ( 15) | 91% ( 41) | 92% ( 36) | 100% ( 39) | 96% ( 24) | 95% ( 37) | 95% ( 40) | 97% ( 28) | 96% ( 27) | 89% ( 16) | 82% ( 18) | 100% ( 18) | 84% ( 21) |
| *Albanian* | 0% ( 0) | 0% ( 0) | 0% ( 0) | 0% ( 0) | 0% ( 0) | 0% ( 0) | 5% ( 2) | 0% ( 0) | 0% ( 0) | 0% ( 0) | 5% ( 1) | 0% ( 0) | 8% ( 2) |
| *Macedonian* | 0% ( 0) | 0% ( 0) | 0% ( 0) | 0% ( 0) | 0% ( 0) | 3% ( 1) | 0% ( 0) | 0% ( 0) | 0% ( 0) | 6% ( 1) | 0% ( 0) | 0% ( 0) | 0% ( 0) |
| *Moroccan* | 0% ( 0) | 2% ( 1) | 0% ( 0) | 0% ( 0) | 4% ( 1) | 0% ( 0) | 0% ( 0) | 0% ( 0) | 0% ( 0) | 0% ( 0) | 5% ( 1) | 0% ( 0) | 0% ( 0) |
| **Main procedure:** |  |  |  |  |  |  |  |  |  |  |  |  |  |
| *Laryngoscopy and other tracheoscopy* | 17% ( 1) | 6% ( 2) | 7% ( 2) | 4% ( 1) | 0% ( 0) | 4% ( 1) | 6% ( 2) | 14% ( 3) | 5% ( 1) | 7% ( 1) | 6% ( 1) | 0% ( 0) | 0% ( 0) |
| *Fiber-optic bronchoscopy* | 0% ( 0) | 6% ( 2) | 4% ( 1) | 4% ( 1) | 0% ( 0) | 12% ( 3) | 12% ( 4) | 19% ( 4) | 5% ( 1) | 7% ( 1) | 11% ( 2) | 21% ( 3) | 12% ( 2) |
| *Other bronchoscopy* | 17% ( 1) | 6% ( 2) | 0% ( 0) | 14% ( 4) | 12% ( 2) | 15% ( 4) | 12% ( 4) | 5% ( 1) | 11% ( 2) | 0% ( 0) | 11% ( 2) | 14% ( 2) | 6% ( 1) |
| *Routine chest x-ray* | 0% ( 0) | 6% ( 2) | 4% ( 1) | 7% ( 2) | 6% ( 1) | 8% ( 2) | 6% ( 2) | 5% ( 1) | 11% ( 2) | 0% ( 0) | 0% ( 0) | 0% ( 0) | 18% ( 3) |
| *Microscopic examination of blood, other microscopic examination* | 0% ( 0) | 0% ( 0) | 0% ( 0) | 4% ( 1) | 6% ( 1) | 0% ( 0) | 0% ( 0) | 10% ( 2) | 0% ( 0) | 20% ( 3) | 6% ( 1) | 14% ( 2) | 0% ( 0) |
| *Removal of intraluminal FB from nose without incision* | 0% ( 0) | 3% ( 1) | 7% ( 2) | 0% ( 0) | 12% ( 2) | 0% ( 0) | 3% ( 1) | 5% ( 1) | 5% ( 1) | 0% ( 0) | 0% ( 0) | 7% ( 1) | 6% ( 1) |
| *Removal of intraluminal FB from pharynx without incision* | 17% ( 1) | 34% (11) | 21% ( 6) | 14% ( 4) | 12% ( 2) | 15% ( 4) | 15% ( 5) | 14% ( 3) | 26% ( 5) | 20% ( 3) | 22% ( 4) | 14% ( 2) | 24% ( 4) |
| *Removal of intraluminal FB from trachea and bronchus without incision* | 17% ( 1) | 6% ( 2) | 21% ( 6) | 25% ( 7) | 6% ( 1) | 12% ( 3) | 9% ( 3) | 5% ( 1) | 5% ( 1) | 7% ( 1) | 17% ( 3) | 0% ( 0) | 0% ( 0) |
| **Secondary intervention (1):** |  |  |  |  |  |  |  |  |  |  |  |  |  |
| *Laryngoscopy and other tracheoscopy* | 0% ( 0) | 0% ( 0) | 0% ( 0) | 5% ( 1) | 0% ( 0) | 0% ( 0) | 9% ( 2) | 7% ( 1) | 0% ( 0) | 22% ( 2) | 20% ( 2) | 10% ( 1) | 20% ( 2) |
| *Fiber-optic bronchoscopy* | 14% ( 1) | 0% ( 0) | 8% ( 1) | 5% ( 1) | 0% ( 0) | 0% ( 0) | 4% ( 1) | 14% ( 2) | 0% ( 0) | 11% ( 1) | 10% ( 1) | 0% ( 0) | 10% ( 1) |
| *Other bronchoscopy* | 14% ( 1) | 0% ( 0) | 0% ( 0) | 10% ( 2) | 0% ( 0) | 6% ( 1) | 4% ( 1) | 7% ( 1) | 0% ( 0) | 0% ( 0) | 10% ( 1) | 0% ( 0) | 0% ( 0) |
| *Routine chest x-ray* | 14% ( 1) | 29% ( 4) | 8% ( 1) | 15% ( 3) | 17% ( 1) | 12% ( 2) | 30% ( 7) | 21% ( 3) | 0% ( 0) | 11% ( 1) | 10% ( 1) | 20% ( 2) | 0% ( 0) |
| *Electrocardiogram* | 0% ( 0) | 14% ( 2) | 8% ( 1) | 5% ( 1) | 0% ( 0) | 0% ( 0) | 9% ( 2) | 0% ( 0) | 20% ( 2) | 11% ( 1) | 10% ( 1) | 10% ( 1) | 10% ( 1) |
| *Microscopic examination of blood, other microscopic examination* | 29% ( 2) | 0% ( 0) | 8% ( 1) | 5% ( 1) | 0% ( 0) | 6% ( 1) | 0% ( 0) | 0% ( 0) | 0% ( 0) | 0% ( 0) | 10% ( 1) | 0% ( 0) | 10% ( 1) |
| *Removal of intraluminal FB from pharynx without incision* | 29% ( 2) | 0% ( 0) | 0% ( 0) | 0% ( 0) | 0% ( 0) | 19% ( 3) | 13% ( 3) | 0% ( 0) | 0% ( 0) | 0% ( 0) | 0% ( 0) | 10% ( 1) | 0% ( 0) |
| *Removal of intraluminal FB from trachea and bronchus without incision* | 0% ( 0) | 7% ( 1) | 15% ( 2) | 5% ( 1) | 33% ( 2) | 6% ( 1) | 4% ( 1) | 7% ( 1) | 10% ( 1) | 0% ( 0) | 0% ( 0) | 10% ( 1) | 0% ( 0) |
